# Nanometronomic treatment of 4T1 breast cancer with nanocaged doxorubicin prevents drug resistance and circumvents cardiotoxicity

**DOI:** 10.18632/oncotarget.14204

**Published:** 2016-12-25

**Authors:** Serena Mazzucchelli, Michela Bellini, Luisa Fiandra, Marta Truffi, Maria A. Rizzuto, Luca Sorrentino, Erika Longhi, Manuela Nebuloni, Davide Prosperi, Fabio Corsi

**Affiliations:** ^1^ Department of Biomedical and Clinical Sciences “Luigi Sacco”, University of Milan, Milan, Italy; ^2^ Department of Biotechnology and Biosciences, University of Milan-Bicocca, Milano, Italy; ^3^ Surgery Department, Breast Unit, ICS Maugeri S. p. A. SB, Pavia, Italy

**Keywords:** metronomic chemotherapy, breast cancer, doxorubicin, drug resistance, tumor targeting

## Abstract

Chemotherapeutic treatment of breast cancer is based on maximum tolerated dose (MTD) approach. However, advanced stage tumors are not effectively eradicated by MTD owing to suboptimal drug targeting, onset of therapeutic resistance and neoangiogenesis. In contrast, “metronomic” chemotherapy is based on frequent drug administrations at lower doses, resulting in neovascularization inhibition and induction of tumor dormancy. Here we show the potential of H-ferritin (HFn)-mediated targeted nanodelivery of metronomic doxorubicin (DOX) in the setting of a highly aggressive and metastatic 4T1 breast cancer mouse model with DOX-inducible expression of chemoresistance. We find that HFn-DOX administered at repeated doses of 1.24 mg kg^−1^ strongly improves the antitumor potential of DOX chemotherapy arresting the tumor progression. We find that such a potent antitumor effect is attributable to multiple nanodrug actions beyond cell killing, including inhibition of tumor angiogenesis and avoidance of chemoresistance. Multiparametric assessment of heart tissues, including histology, ultrastructural analysis of tissue morphology, and measurement of markers of reactive oxygen species and hepatic/renal conditions, provided evidence that metronomic HFn-DOX allowed us to overcome cardiotoxicity. Our results suggest that HFn-DOX has tremendous potential for the development of “nanometronomic” chemotherapy toward safe and tailored oncological treatments.

## INTRODUCTION

Over the past decades, cytotoxic chemotherapeutics have dominated the systemic management of cancer according to the “maximum tolerated dose” (MTD) paradigm [1, 2]. MTD therapy requires that patients are administered with single dose or short courses of the highest tolerable dosage of a drug in order to achieve the best therapeutic efficacy. Due to low tumor selectivity, MTD treatments cannot be protracted in order to allow recovery of healthy tissues and to reduce myelosuppression associated with pulsed drug doses [3]. In fast-growing or metastatic tumors, during these therapeutic breaks, a burst in cancer cell proliferation accompanied by manifestation of chemoresistance and accelerated angiogenesis are likely [4, 5]. Hence, a reappraisal of advanced-stage cancer management is ongoing, moving from the “maximum tolerable” to the “minimum effective” dose paradigm [6]. Indeed, cytotoxic agents administered at low dosages are expected to allow protracted treatments and have been suggested to up-regulate antiangiogenic factors such as thrombospondin-1 and to inhibit vascular endothelial growth factor and platelet-derived growth factor [2, 7].

The first clinical trials using low-dose metronomic (LDM) chemotherapy were conducted for breast, prostate, gastrointestinal, renal and pancreatic cancers, as well as refractory melanoma [8–10]. This regimen is based on a lower dose of drugs administered more frequently, without the need of extensive interruptions [2, 11]. While the conventional dose-dense chemotherapeutic setting is suggested to act by targeting the proliferating tumor cells [12], LDM is presumed to affect the vasculature growth and repair [8, 13], to reduce systemic toxicity and myelosuppression, and to improve the stimulation of the host immune system against the tumor [1, 4]. However, several limiting factors remain for LDM in order to displace MTD treatments in clinical practice, including 1) low drug accumulation at tumor site [14], 2) controversial effectiveness against chemoresistance in advanced metastatic cancers [15], and 3) acquired resistance after prolonged treatment [16].

Recent advances in nanotechnology could offer groundbreaking solutions to improve the effectiveness of LDM chemotherapy, by taking advantage of the unique targeting efficiency of engineered nanocarriers [17]. In the present work, we propose a new concept of low dose “nanometronomic” (LDNM) chemotherapy. In principle, it is possible to obtain a prolonged antitumor effect with LDNM by means of multitasking nanocarriers that deliver lower dose of drug selectively to the growing tumor, inhibit the neovascularization process and prevent chemoresistance. Doxorubicin (DOX) is an excellent pilot drug for use in a LDM regimen [18], as its great anticancer efficacy is notoriously dose-limited by severe systemic side effects above all long-term cardiotoxicity with different severity grades from reduction in left ventricular ejection fraction (LVEF) to severe congestive heart failure [19, 20]. Liposomal anthracyclines, including pegylated liposomal doxorubicin (pl-DOX), have been introduced in clinical practice to enhance the therapeutic index and to avoid cardiotoxicity of these drugs thanks to higher accumulation of DOX in the tumor with reduced concentration in off-target organs [21]. However, meta-analyses of several clinical trials comparing pl-DOX to conventional DOX have demonstrated reduced (but not annulled) cardiotoxicity of pl-DOX, without improvement in progression-free or overall survival in advanced breast cancer (BC) [22]. Therefore, improving the therapeutic index of DOX remains an open challenge. As an ideal DOX nanocarrier for our LDNM study, we used H-Ferritin (HFn) nanocages, recently proposed as a promising bionanoparticle for cancer targeting [23] owing to its affinity for transferrin receptor 1 (TfR-1), which is constitutively overexpressed in primary and metastatic cancer cells [24]. HFn-DOX complex was recently demonstrated to overcome chemoresistance by actively promoting DOX nuclear translocation *in vitro* [25, 26] and was tested as a MTD treatment of a DOX-sensitive BC animal model with encouraging results [27].

## RESULTS

### *In vitro* uptake and cytotoxicity of HFn-DOX in 4T1 breast cancer cells

**Figure 1:**
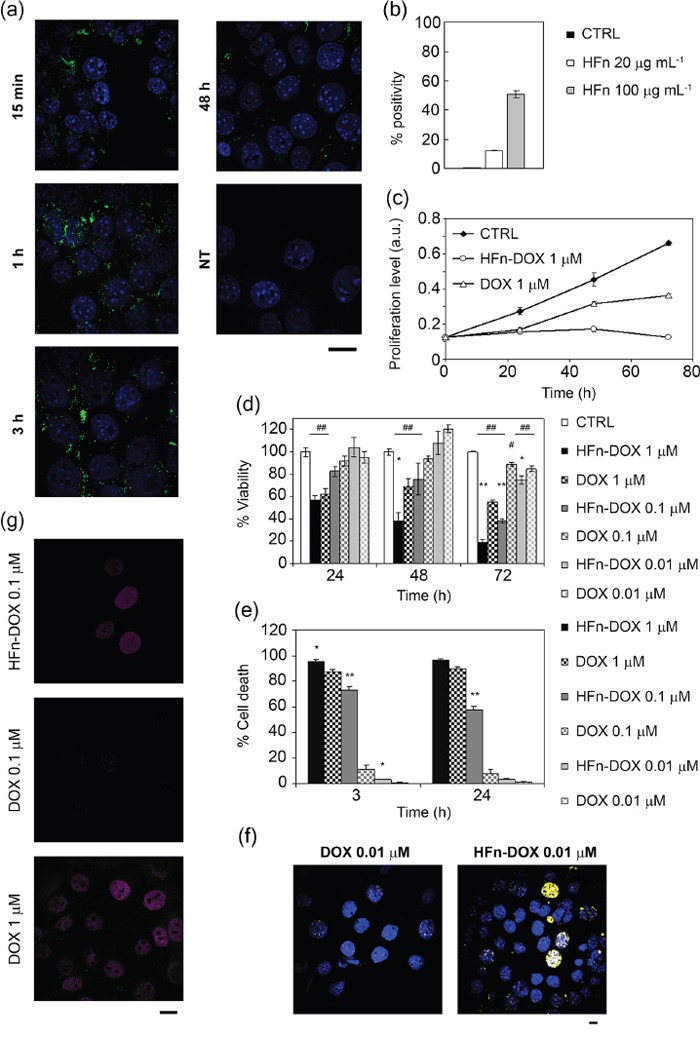
*In vitro* activity of HFn-DOX nanocages toward tumor cells **a.** Intracellular localization of HFn nanoparticles. Confocal microscopy merged images of 4T1-L cells, incubated for 15 min, 1, 3 and 48 h at 37 °C with 100 μg mL−1 of FITC-labeled HFn (green). Nuclei were stained with DAPI (blue). Scale bar: 10 μm. **b.** HFn binding toward 4T1-L breast cancer cells. 4T1-L cells were incubated 2 h at 4 °C with FITC-labeled HFn (20 and 100 μg mL−1) and then processed for flow cytometry. Untreated cells were used as control to set the positive region. **c.** Proliferation profiles of cells treated with 1 μM DOX or HFn-DOX for up to 72 h. Untreated cells are used as control. Values are mean of six replicates ± SE. **d.** Viability of cells treated with free or nanoformulated DOX. 4T1-L cells were treated with 1, 0.1, and 0.01 μM DOX or HFn-DOX for up to 72 h. Viability was assessed by measuring the conversion of MTT into formazan, normalized on cell proliferation of untreated cells. Statistical significance vs. CTRL #P<0.05, ##P<0.005; Statistical significance vs. DOX *P<0.01; **P<0.005. **e.** Cell death assay using DOX or HFn-DOX. 4T1-L cells were treated with 1, 0.1, and 0.01 μM DOX or HFn-DOX for 3 or 24 h. Cell death was assessed on the basis of the exposure to Annexin V, evaluated by flow cytometry. Untreated cells were used to set region of positivity. Values are mean of three replicates ± SE. Statistical significance vs. DOX *P<0.005; **P<0.0005. **f.** Double-strand break of DNA after DOX exposure. Confocal microscopy images of 4T1-L cells incubated with 0.01 μM DOX or HFn-DOX. Anti-γH2A.X antibodies were used to reveal the DNA double-strand breaks (DSB; yellow). Nuclei were stained with DAPI (blue). Scale bar: 10 μm. **g.** Doxorubicin release inside the nuclear compartment. Confocal microscopy images of 4T1-L cells incubated with 0.1 μM DOX or HFn-DOX and with 1 μM DOX for 3 h at 37 °C. DOX signal is represented in magenta, while DOX degradation product in green. Scale bar: 10 μm.

The 4T1 cell line (4T1-L) was selected as *in vitro* and *in vivo* BC model for three main reasons: 1) tumor aggressiveness due to 4T1 genetic patterning, which results in high level of proliferation, migration and invasiveness; 2) basal expression of MDR-1 transporter, which switches into overexpression upon treatment with DOX resulting in chemoresistance [28]; 3) stable luciferase expression, which allowed us to follow the tumor progression and metastases. 4T1-L cells were first treated with FITC-labeled HFn (FITC-HFn) [25] to investigate the nanoparticle-cell interaction. Cells were incubated with FITC-HFn for 15 min, 1, 3 and 48 h, and analyzed by confocal microscopy to evaluate the uptake and intracellular trafficking. HFn was quickly internalized, since it was recovered inside the cell cytoplasm after only 15 min of incubation, and it continued accumulating in the cytosol until 3 h (Figure 1A). The intracellular signal intensity decreased after 48 h probably due to ferritin disassembly, consistent with previous evidence [25]. HFn was found partly compartmentalized in early endosomes and partly free in the cytosol (Supplementary Figure 1), while the absence of colocalization with lysosomes, Golgi and transferrin (Tf) marker suggested that HFn did not follow lysosomal degradation, elimination or recycling, respectively, in agreement of previous evidence [25]. Binding assays with HFn at 20 or 100 μg mL^−1^ confirmed a dose-dependent recognition of tumor cells (Figure 1B).

4T1-L cells were treated with DOX or HFn-DOX at increasing concentrations of DOX to assess cell proliferation, cell death, DNA damage and nuclear DOX accumulation. Proliferation was arrested for at least 72 h after treatment with 1 μM HFn-DOX, while DOX reduced cell proliferation for 24 h only, suggesting the onset of chemoresistance upon incubation with DOX (Figure 1C). Cell viability was evaluated by incubating the cells with 0.01, 0.1 and 1 μM DOX or HFn-DOX for up to 72 h. Results reported in Figure 1D show that inhibition of BC cell viability using HFn-DOX was significantly higher than that after treatment with DOX. Such a drop in viability was ascribed to a remarkable increase in cell death (Figure 1E). Treatment with 0.01 μM HFn-DOX caused pronounced apoptosis and necrosis induction and double strand breaks in contrast to DOX (Figure 1F). It can be assumed that the increase in cytotoxicity of HFn-DOX resides in the efficiency of HFn in promoting DOX nuclear translocation (Figure 1G), as already described for different tumor cell lines [25, 26]. Quantitative fluorescence analysis of confocal images gave a nuclear DOX concentration of 15.2 and 9-fold higher than that detected in cultures treated with DOX at 0.1 and 1 μM, respectively (Supplementary Figure 2).

### *In vivo* targeting and biodistribution of HFn nanocarrier

**Figure 2:**
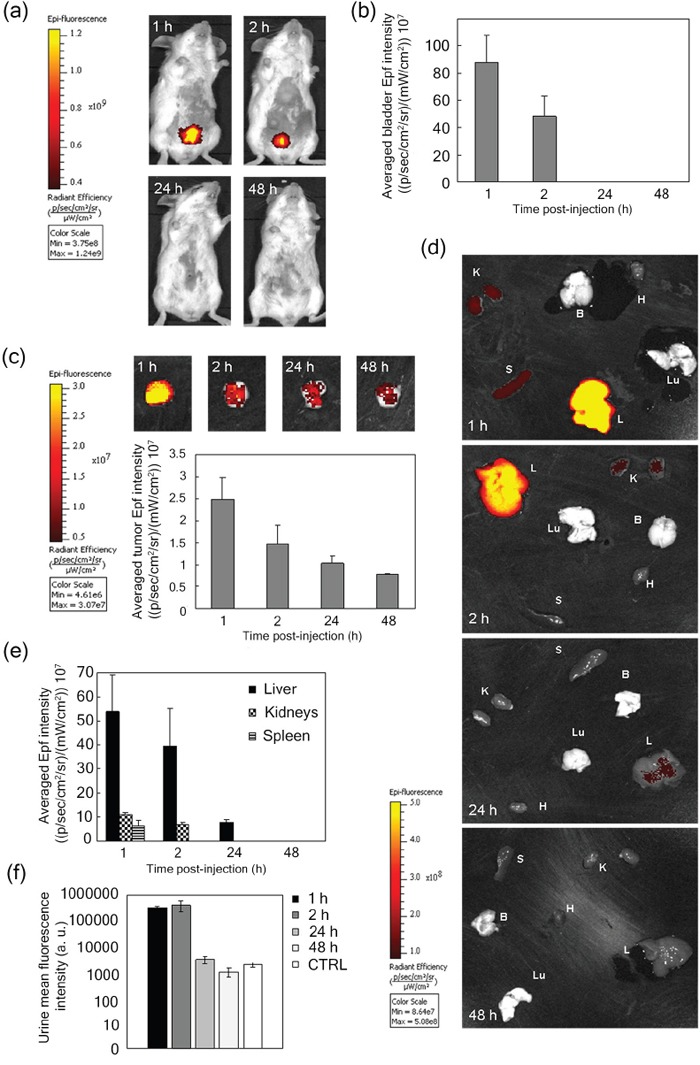
*In vivo* targeting and distribution of void HFn nanocarrier **a.** Epifluorescence (Epf) images of mice bearing 4T1-L tumors acquired 1, 2, 24 and 48 h after intravenous (i.v.) injection into the tail vein of 5 μg kg−1 AF660-HFn and **b.** averaged Epf intensity of the bladder region of interest (ROI). **c.** Epf of isolated 4T1 tumors and averaged Epf intensity of tumor ROI acquired 1, 2, 24 and 48 h after exposure to HFn. **d.** Epf of isolated spleen (S), kidneys (K), liver (L), brain (B), heart (H), lungs (Lu), and **e.** averaged Epf intensity of the ROI obtained after 1, 2, 24 and 48 h exposure to HFn. **f.** Fluorescence intensity of urine collected 1, 2, 24 and 48 h after i.v. injection of AF660-HFn. The color scale in panels a, c and d indicates the averaged epifluorescence expressed as radiant efficiency [(p/sec/cm2/sr)/(mW/cm2)], where p/sec/cm2/sr is the number of photons per second that leave a square centimeter of tissue and radiate into a solid angle of one steradian (sr). Values reported in panels b, c, e and f are mean ± SE of at least 4 different samples under each experimental condition.

An orthotopic 4T1 metastatic BC model was obtained by implanting 4T1-L cells (10^5^ cells) subcutaneously in the mammary fat pad of female Balb/C mice [29]. This murine tumor was reported to metastasize primarily, yet not exclusively, by a hematogenous route leading to metastatic spread to lung, liver and lymph nodes [30]. The reliability of the model was confirmed by following tumor progression and early onset of metastases by bioluminescence intensity (BLI) imaging over 20 days (Supplementary Figure 3A). Histopathological analysis performed on excised tumors confirmed that the primary mass was indeed derived from epithelial cancer cells without undesired morphological alterations (Supplementary Figure 3B). 4T1 mice were injected into the tail vein with Alexa Fluor_660_-labeled HFn (AF660-HFn) at 5 μg kg^−1^ [31] and monitored by live fluorescence imaging at 1, 2, 24 and 48 h. An intense epifluorescence signal (Epf) at the bladder was detected within the first 2 h, which however disappeared after 24 h (Figure 2A and 2B), suggesting renal excretion of HFn within 1 day. Epf of excised tumors 1, 2, 24 and 48 h after AF660-HFn injection displayed rapid tumor uptake, which progressively decreased in intensity over time (Figure 2C). Confocal images acquired on cryosections of excised tumors confirmed that HFn reached the 4T1 cell cytoplasm and thus were not confined to the tumor stroma or vessels, but actively entered into cancer cells (Supplementary Figure 4). Combined data reported in Figure 2A-2C suggested that a prevalent fraction of nanoparticles that were not captured by the tumor were rapidly sequestered by the kidneys, and presumably eliminated into the bladder. This hypothesis was confirmed by Epf analysis of excised kidneys that exhibited a detectable AF660-HFn fluorescence emission at 1 and 2 h (Figure 2D and 2E) and further evidence was provided by fluorescence measurement of collected urine (Figure 2F). Besides kidney filtration, our results suggested preferential distribution of HFn in the liver within the first 24 h and appreciable Epf was also detected in the spleen for up to 2 h. In contrast, HFn were not recovered in the lungs, heart and brain (Figure 2D and 2E).

### Bioavailability of HFn-DOX and accumulation at the tumor

**Figure 3:**
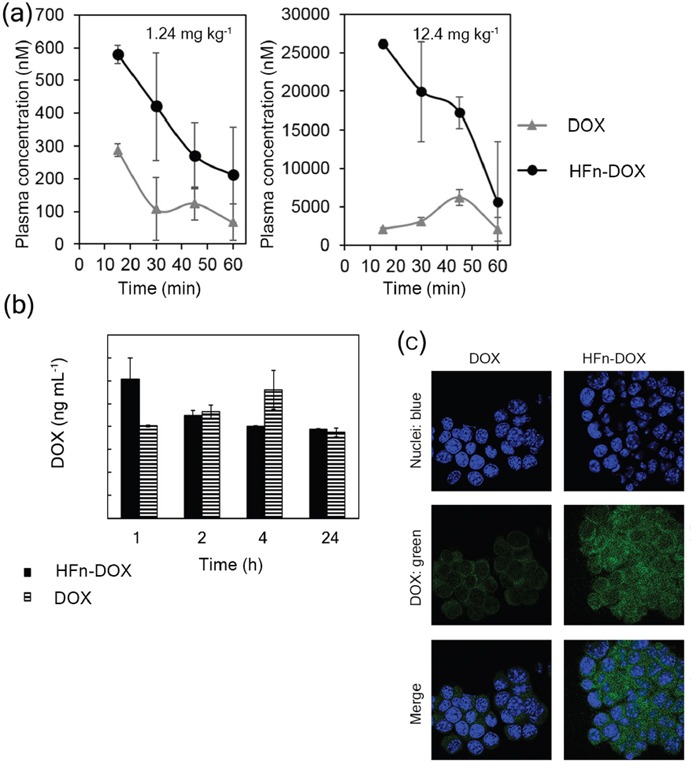
Bioavailability and tumor accumulation of HFn-DOX in comparison with free DOX **a.** Bioavailability of DOX and HFn-DOX at different time points. Plasma concentration of DOX after i.v. injection of HFn-DOX (black circles) or DOX (gray triangles) at 1.24 mg kg−1 and 12.4 mg kg−1 in healthy mice. **b.** DOX accumulation at 4T1-L tumor in mice at different time points after administration of 1.24 mg kg−1 DOX as free molecule or HFn-DOX. Female Balb/C mice orthotopically implanted with 4T1-L murine mammary carcinoma cells were injected 6 days after implantation (time 0) with DOX or HFn-DOX. DOX levels in tumor have been determined 1, 2, 4, and 24 h after i.v. injection following acidified isopropanol extraction from tumor homogenates. Aliquots from six mice per each time point concentration have been extracted and analyzed by spectrofluorimeter. Reported values are means of 3 samples/group ± SE. P values are summarized in Table S5. **c.** Confocal microscopy images of 4T1-L cells dissociated from tumor harvested 2 h after i.v. injection of DOX and HFn-DOX. DOX signal is represented in green, while nuclei were stained with DAPI (blue). Scale bar: 10 μm.

To evaluate the bioavailability of nanoformulated drug, 2 groups of healthy mice (5 mice/group) were treated with DOX or HFn-DOX at 1.24 mg kg^−1^. Blood samples were collected from the retro-orbital plexus at 15, 30, 45 and 60 min. These tight time points were chosen to detect possible changes in blood bioavailability of DOX or HFn-DOX soon after administration, as in both cases the drug was injected intravenously. DOX was extracted from collected samples and quantified by fluorescence intensity analysis (FLI) at λ_em_ = 550 nm (λ_ex_ = 500 nm) [32]. Blood samples taken before drug administration were set as reference. Bioavailability of HFn-DOX was two-fold higher than DOX although the kinetic seems to maintain the same shape. To better discriminate kinetic's variations due to nanoformulation, a 10-fold higher dosage of DOX or HFn-DOX (i.e. 12.4 mg kg^−1^) has been administered to healthy mice. Results reported in Figure 3A display different plasma distribution profiles and confirming that HFn-DOX increases drug bioavailability in comparison to DOX of at least four-fold at each time point

Accumulation of DOX at primary tumors (Figure 3B) was determined by fluorescence after chemical extraction from homogenates of resected tumors at 1, 2, 24, and 48 h after single injection of HFn-DOX or DOX at 1.24 mg DOX kg^−1^ [33]. DOX was found in higher concentration in tumors of mice treated with HFn-DOX compared to DOX within 1 h. HFn-DOX displayed faster localization at the tumor compared to DOX, (Figure 3B), suggesting a crucial role for nanoparticle-mediated delivery in enhancing the tumor targeting, although after 2 h the DOX levels are equilibrated in both cases. Confocal images of 4T1-L dissociated from tumors excised at 2 h evoked higher tumor cell accumulation of DOX in samples treated with HFn-DOX compared to DOX (Figure 3C). Combining these results suggested that HFn-DOX were efficiently captured by tumor cells, while DOX was confined in blood vessels of the tumor to a much larger extent.

### Impact of LDNM monotherapy on breast cancer management

**Figure 4:**
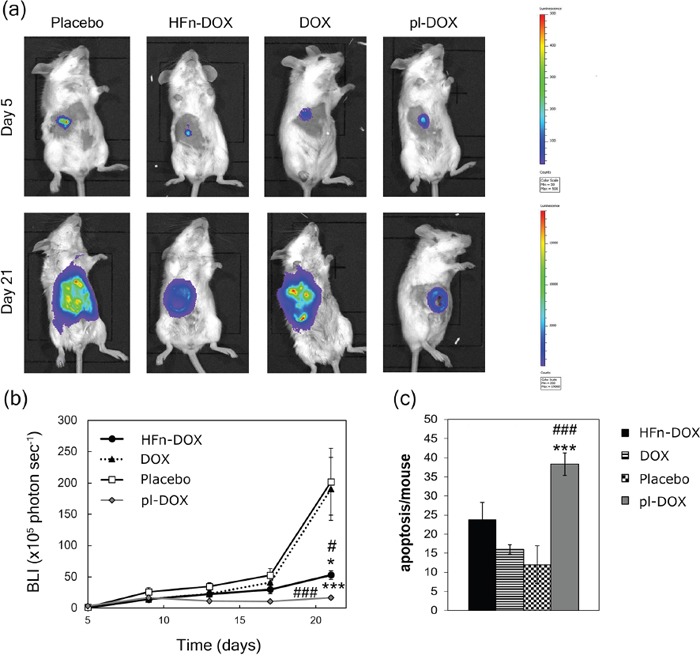
Efficacy of LDNM treatment with HFn-DOX **a.** DOX and HFn-DOX in vivo efficacy. Bioluminescence imaging of female Balb/C mice (n = 12/group) orthotopically implanted (day 0) with 4T1-L murine mammary carcinoma cells were treated with placebo or with 1.24 mg kg−1 of DOX, pl-DOX or HFn-DOX. Drug injections were performed into the tail vein at day 5, 9, 13 and 17. Mice were sacrificed at day 21. **b.** Quantification of tumor volume. Tumor volume was quantified by measuring the bioluminescence intensity signal of 4T1-L cells 5 min after intraperitoneal injection of luciferin. Dots represent the normalized mean value of BLI tumor signal ± SE. Statistical significance vs. placebo #P<0.05 ###P<0.005; vs. DOX *P<0.05 ***P<0.005. **c.** Quantification of apoptosis in tumor tissue upon treatment with HFn-DOX. Tumors excised at day 21 (n = 6/group) were fixed with formalin and embedded in paraffin. Histological slides were processed to label DNA fragments of apoptotic cells. Reported values are the mean of apoptotic cells number/field/sample ± SE. The count was performed on 10 fields/sample. Magnification 20×. Statistical significance vs. placebo ###P<0.005; vs. DOX ***P<0.005.

Eight-week old Balb/C female mice were implanted with 4T1-L cells at day 0. Tumor-bearing mice were randomly divided into three experimental groups at day 5 and treated with placebo, DOX, pl-DOX or HFn-DOX under our LDNM setting: drug administration (1.24 mg DOX kg^−1^) was performed at day 5, 9, 13 and 17. The progression of tumor volume was monitored *in vivo* before each individual drug injection by bioluminescence imaging. Images suggested that HFn-DOX could decrease tumor growth and metastatic spread (Figure 4A and Supplementary Table 1). Indeed, while DOX displayed a tumor progression similar to the control along the experimental window (Figure 4B), HFn-DOX could suppress the tumor growth as long as the drug was administered (day 17) and exhibited a prolonged effect up to the experimental endpoint (day 21). An even better effect was achieved with pl-DOX, which was indeed able to arrest the tumor development. Immunohistochemical analysis of tumor sections showed that the apoptotic effect of pl-DOX and HFn-DOX on BC cells was better than that of DOX (Figure 4C and Supplementary Figure 5), presumably due to the improved tumor accumulation of the drug. However, the absence of statistical significance in apoptosis between HFn-DOX and DOX treated samples advocated alternative factors in the stronger antitumor efficacy of HFn-DOX beyond mere cytotoxicity.

### Impact of LDNM regimen on tumor angiogenesis and chemoresistance

**Figure 5:**
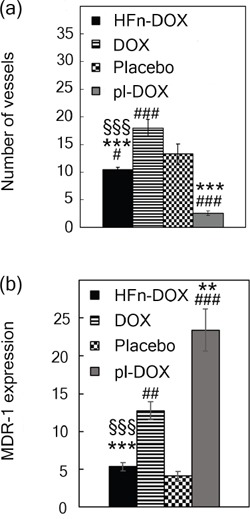
Impact of LDNM regimen on tumor angiogenesis and chemoresistance **a.** Quantification of angiogenesis in tumor tissue upon treatment with HFn-DOX. Tumors excised at day 21 (n = 5/group) were fixed with formalin and embedded in paraffin. Immunohistochemistry of histological slides were processed to label CD31+ cells. Reported values are mean of vessel number counted in 10 fields/sample ± SE. Magnification 40×. Statistical significance vs. placebo #P<0.005 ###P<0.00005; vs. DOX ***P<0.00005; vs. pl-DOX §§§P<0.0005. **b.** Quantification of MDR-1-expression. Excised tumors (n = 5/group) were processed for immunohistochemistry of MDR-1 antigen. The percentage of image area positive for MDR-1 expression was quantified using ImagePro Plus Software. Reported values are the mean of the percentage of MDR-1 positive signal counted in 5 fields/sample ± SE. Statistical significance vs. Placebo ##P<0.0005 ###P<0.00005; vs. DOX **P<0.005 ***P<0.0005; vs. pl-DOX §§§P<0.0005.

In line with the observed discrepancy in the results from the DNA fragmentation assay (Figure 4C), we investigated the possible involvement of anti-angiogenic effect of HFn-DOX under the LDNM regimen. Vessel labeling with anti-CD31 antibody in BC histological slides revealed a remarkable decrease in the number of CD31-positive cells compared to DOX (Figure 5A and Supplementary Figure 6), suggesting a role of the HFn-DOX-promoted antiangiogenic effect on the inhibition of tumor progression and diffusion. Analogously, it is likely that the strong inhibition in tumor growth observed with pl-DOX (Figure 4B) was primarily due to anti-angiogenic activity (Figure 5A). 4T1-L BC cells have been described to develop drug resistance owing to induced overexpression of MDR-1 protein upon standard treatment with DOX [28]. Western blot performed on 4T1 cells treated for 72 h with 0.1 μM DOX corroborated DOX induction of MDR-1 expression *in vitro* (Supplementary Figure 7). We examined MDR-1 expression in tumor tissues dissected after LDNM treatment. Tumor sections from DOX and pl-DOX-treated mice displayed a three-fold and five-fold increase in MDR-1-positive cells, respectively, compared to animals treated with placebo and HFn-DOX treated animals (Figure 5B and Supplementary Figure 8). As expected, both DOX and pl-DOX induced an obvious overexpression of MDR-1 in tumor cell membranes, which was particularly pronounced in the proximity of the tumor endothelium [34]. In contrast, MDR-1 expression was undetectable in tumor cell membranes after HFn-DOX treatment and was found only to a limited extent in tumor vessels after HFn-DOX treatment, at the same level of the placebo. This result is relevant in view of a protracted metronomic treatment preventing the onset of chemoresistance, and it is even more surprising considering that pl-DOX is commonly used in patients previously treated with anthracyclines and therefore affected by potentially chemoresistant cancers.

### HFn-DOX suppresses DOX cardiotoxicity and systemic dysfunction under a LDNM therapeutic setting

**Figure 6:**
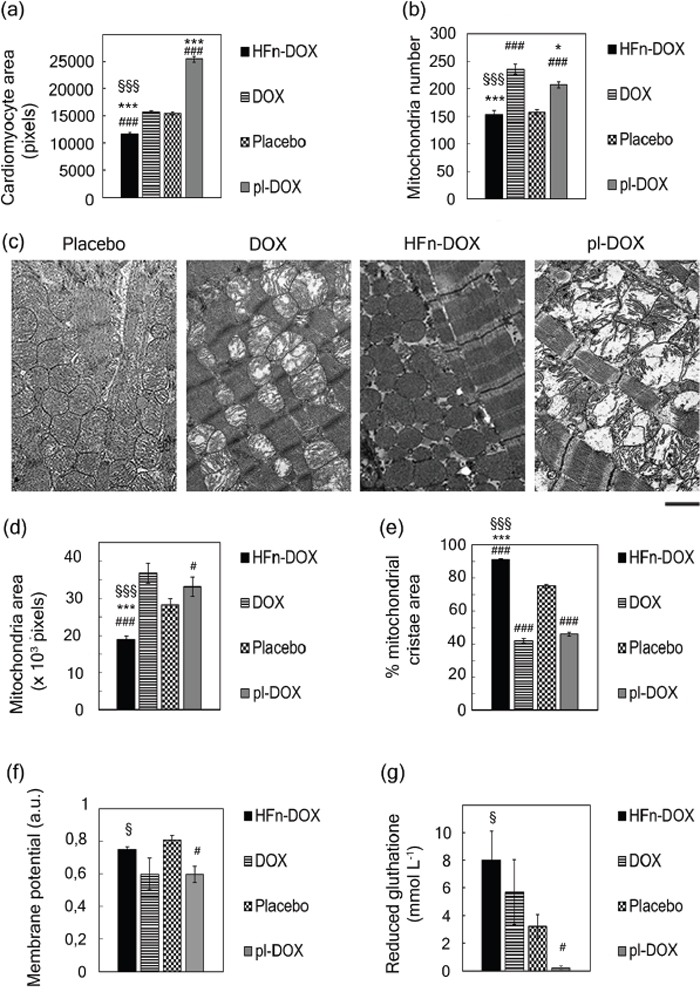
Examination of cardiotoxicity of HFn-DOX, pl-DOX and DOX **a.** Nanodelivery protects cardiomyocytes from DOX-induced hypertrophy. Hearts excised at day 21 (n = 3/group) from mice treated with placebo or with 1.24 mg kg−1 of DOX, pl-DOX or HFn-DOX were fixed with formalin and embedded in paraffin. Histological slides of cardiac sections stained with FITC-WGA were analyzed with ImageJ software to measure cross-section area of cardiomyocytes. Quantification was performed on at least 5 images/group, reporting the mean value of cross-section area of 250 cells/group± SE. Statistical significance vs. Placebo ###P<0.00005; vs. DOX ***P<0.00005; vs. pl-DOX §§§P<0.00005. **b.** Nanodelivery protects against DOX-induced mitochondrial toxicity. Hearts excised at day 21 (n = 3/group) from mice treated with placebo or with 1.24 mg kg−1 of DOX, pl-DOX or HFn-DOX were fixed with glutaraldehyde and embedded in epoxy-resin. TEM images of ultrathin heart sections of cardiac tissues acquired at 4200 magnifications were analyzed with ImageJ to measure the number of mitochondria in heart tissue. Quantification was performed on at least 9 images/group, reporting the mean mitochondria number/image ± SE. Statistical significance vs. Placebo ###P<0.00005; vs. DOX ***P<0.00005 *P<0.005; vs. pl-DOX §§§P<0.00005. **c.** Representative images of hearts excised at day 21 (n = 3/group) from mice treated with placebo or with 1.24 mg kg−1 of DOX, pl-DOX or HFn-DOX. TEM images of ultrathin heart sections of cardiac tissues have been acquired at 11500 magnifications. **d.** Nanodelivery reduces the mitochondrial size growth due to DOX treatment. Quantification of TEM images (ImageJ) of ultrathin heart sections acquired at 11500 magnifications. Quantification was performed on at least 10 images/group, measuring at least 100 mitochondria/sample. Values represent the mean mitochondrial area ± SE. Statistical significance vs. Placebo ###P<0.00005 #P<0.05; vs. DOX ***P<0.00005; vs. pl-DOX §§§P<0.00005. **e.** Nano delivery limits the damage of mitochondrial cristae from DOX. Quantification of TEM images (Image J) of ultrathin heart sections acquired at 11500 magnifications. Quantification was performed on at least 10 images/group, measuring at least 100 mitochondria/sample. Values represent the percentage of mitochondrial area occupied by cristae ± SE. Statistical significance vs. Placebo ###P<0.00005; vs. DOX ***P<0.00005; vs. pl-DOX §§§P<0.00005 **f.** HFn-DOX does not affect mitochondrial membrane potential. Mitochondrial membrane potential was measured by staining-isolated mitochondria from mouse heart tissue dissected at day 21 (n = 3/group) from mice treated with placebo or with 1.24 mg kg−1 of DOX, pl-DOX or HFn-DOX. Statistical significance vs. Placebo #P<0.05; vs. pl-DOX §P<0.05. **g.** HFn-DOX does not decrease the concentration of reduced GSH. The extent of reduced GSH was measured in lysates of hearts excised at day 21 (n = 3/group) from mice treated with placebo or with 1.24 mg kg−1 of DOX, pl-DOX or HFn-DOX. Values represent the mean GSH concentration in heart extracts± SE. Statistical significance vs. Placebo #P<0.05; vs. pl-DOX §P<0.05.

Cardiotoxicity represents a life-threatening unresolved issue associated to DOX chemotherapy under clinically relevant settings [35]. To evaluate the incidence of LDNM monotherapy on cardiotoxicity, we followed a multiparametric approach [36]. First, histological slides of heart tissues were treated with FITC-conjugated wheat germ agglutinin (FITC-WGA), a cell membrane label, and imaged by fluorescence microscopy (Supplementary Figure 9). Cardiomyocyte cross-sections from mice treated with HFn-DOX, pl-DOX, DOX or non-treated were measured at day 21. Images showed a significant increase of cardiomyocyte area in DOX and pl-DOX samples suggesting a strong cellular damage response compared to HFn-DOX samples (Figure 6A). Detailed ultrastructural analysis of cardiac cells in DOX and pl-DOX treated samples revealed an increased number of mitochondria compared to HFn-DOX (Figure 6B and Supplementary Figure 10). In addition, changes in mitochondria morphology, including larger surface area and cristae depletion, typical effects of DOX-induced cardiomyopathy [36], were clearly evident in DOX and pl-DOX treated samples but not in HFn-DOX samples (Figure 6C-6E). Therefore, the absence of obvious alterations in mitochondria number and morphology in heart samples from mice treated with HFn-DOX strongly supports the lack of cardiotoxicity in LDNM HFn-DOX treatment, even compared to pl-DOX, which is currently considered the most safe anthracycline therapy in terms of cardiotoxicity.

To further investigate if the ultrastructural alterations were associated to mitochondrial dysfunction, mitochondria isolated from heart tissue of DOX, pl-DOX or HFn-DOX treated mice were analyzed in detail. The membrane potential decreased by 30% in DOX and pl-DOX samples compared to HFn-DOX (Figure 6F). As mitochondrial impairment was expected to generate reactive oxygen species (ROS) [37], we quantified the level of the ROS quencher glutathione (GSH) in heart tissue [38]. Figure 6G displays the lower amount of reduced GSH in DOX and pl-DOX-treated mice in comparison to HFn-DOX, confirming mitochondrial dysfunction induced by treatment with free and liposomal DOX only.

Finally, we assessed the systemic toxicity of HFn-DOX by histopathological examination of liver, kidneys, lung, spleen, heart, gut and brain isolated at day 21. No histological lesions were found in all organs (Supplementary Figure 11). Liver and kidney functionalities were also determined to further evaluate the toxicity profile of HFn-DOX treatment. Serum levels of aspartate transaminase (AST) and alanine transaminase (ALT) (Supplementary Table 2), and urea and creatinine (Supplementary Table 3), were monitored as markers of liver and kidney condition, respectively. Our results showed that AST/ALT and urea/creatinine ratios in HFn-DOX treated mice were comparable to the control and in the range of reference, confirming the overall nanodrug safety.

## DISCUSSION

In the present study a highly aggressive metastatic BC model based on murine 4T1 cells was established. This allowed us to simulate the dramatic clinical picture of advanced BC patients and to evaluate the impact of DOX nanoformulation under LDM monotherapy in compromised subjects, as DOX remains a mainstay therapy in various solid tumors. Our results suggest that DOX monotherapy does not affect tumor progression significantly: although the expected cytotoxicity was confirmed *in vitro*, this did not translate into substantial antitumor activity *in vivo* in an advanced-stage BC model, whereas off-target tissue accumulation and myocardial damage largely occurred. This result is reminiscent of the frustrating clinical condition in which chemotherapy fails to overcome BC progression and combination therapies become necessary to control the disease. Therefore, the potential of LDM DOX to overcome the limitations of dose-dense regimens in advanced-stage tumors remains questionable, since DOX requires high doses to gain a proper drug concentration at cancer deposits [39–40]. In contrast, the results of our study demonstrate that our LDNM strategy, which combines LDM administration of DOX with HFn-delivery resulted in a targeted effect of DOX on 4T1 cancer cells together with a sustained antiangiogenic activity in the tumor microenvironment. Indeed, HFn-DOX exhibited potent antitumor activity when administered at frequent doses as low as 1.24 mg kg^−1^ *in vivo* compared to free DOX and placebo. Such a strongly improved activity correlates with the pharmacokinetic profile of LDNM DOX, as emerged from a recent biodistribution study [41]. Indeed, while DOX displayed reduced bioavailability, high levels of HFn-DOX were recovered in plasma during the first few hours post-injection that were attributable to a lower sequestration by off-target organs [41]. HFn-DOX could accumulate in the tumor site exploiting the EPR effect [42] or by endothelial wall transcytosis promoted by TfR-1 recognition [43] and it is internalized in tumor cells by receptor-mediated endocytosis [44]. HFn-mediated target selectivity conferred earlier intra-tumor activity to the drug, lower off-target accumulation with fast liver metabolism and rapid clearance of circulating excess drug by renal excretion, suggesting optimal therapeutic index in future clinical translation [41, 44]. Beyond its favorable bioavailability and target selectivity, a plausible explanation for enhanced antitumor activity of LDNM HFn-DOX resides in HFn propensity to behave like a Trojan horse, imparting DOX with drastically enhanced nuclear penetration even in resistant cancer cells [25, 45]. Such HFn property can greatly improve current strategies of LDM chemotherapy, due to sustained nuclear release of a DNA-damaging drug. Indeed, our *in vitro* experiments showed that HFn-mediated delivery allowed a 15.2-fold increase of DOX nuclear concentration within 3 h as compared to the drug alone.

Even drug resistance significantly impacts on BC management, accounting for a relevant proportion of patients in which anthracycline therapy fails to persistently eradicate the tumor [46]. MDR-1 protein is one of the most active multidrug resistance mediators in BC and it is gradually overexpressed under DOX chemotherapy regimens [28]. Negligible MDR-1 induction in tumor cells *in vivo* after LDNM DOX administration suggested that the multidrug resistance machinery of BC cells did not “sense” the cytotoxic agent in HFn-DOX. Otherwise, the dramatic increase of MDR-1 expression observed in samples from mice treated with DOX and even with pl-DOX, suggested that LDNM administration associated with cell nuclear targeting could circumvent DOX resistance dependent by MDR-1.

The general assumption that LDM therapy is essentially due to inhibition of angiogenesis, rather than directly killing residual cancer cells [8], should be reconsidered in the framework of LDNM regimen, in which a key role of targeted action could be reappraised. Combining our data from angiogenesis inhibition with the results from tumor progression (i.e BC growth curves and DNA fragmentation assay) we concluded that targeted action of HFn-DOX on BC cells and antiangiogenic effect of LDNM regimen could play a synergistic role in the increased antitumor efficacy of HFn-DOX compared to DOX alone.

Importantly, LDNM chemotherapy exhibited a safe toxicity profile, as proven by apparent lack of systemic side effects. This is expected to have great clinical impact because cardiotoxicity and general side effects lead to major restriction in the clinical use of anthracyclines. HFn-DOX was less cardiotoxic compared to DOX and even to pl-DOX, although the latter has been associated with improved cardiac safety in various clinical studies. Nevertheless, myocardial alterations provoked by pl-DOX (Figure 6) are not surprising. Indeed, a certain degree of myocardial damage has been previously demonstrated in endomyocardial biopsies of patients treated with pl-DOX, and ultrastructural damage of pl-DOX has not been explored [38, 47]. Moreover, although pl-DOX is less cardiotoxic, it does not significantly reduce relevant cardiac events, and a clinician's preference for pl-DOX over conventional DOX to avoid clinically significant cardiac events is not justified in patients without concurrent cardiac disorders that were not previously subjected to anthracycline exposure [48]. Therefore, the general confidence on low cardiotoxicity of pl-DOX should be reconsidered in the light of these considerations. Otherwise, LDNM treatment with HFn-DOX didn't display anthracycline-related cardiotoxicity, even in comparison with pl-DOX, and it is therefore a promising option for anthracycline therapeutic regimens in cardiosensitive subjects.

We acknowledge a potential limitation relating to immunogenicity in clinical translation of HFn-DOX [49]. Although it is difficult to predict the long-term effect of prolonged treatments in humans, we have collected preliminary data suggesting negligible immunogenicity of HFn in animals. Another limitation of the study is the experimental timespan limited to three weeks. However, based on our findings we could postulate that after 21 days the metronomic treatment by HFn-DOX would lead to further reduction of cancer deposits, as expected by the excellent cytotoxicity showed by HFn-DOX *in vitro*. Moreover, the fact that MDR-1 expression remained stable over time upon treatment with HFn-DOX suggests avoidance of chemoresistance, thus a sustained anticancer activity even after 21 days is expected. About cardiotoxicity, our findings suggest that substantially no myocardial damage is present after treatment with HFn-DOX, and we should expect the same lack in cardiotoxicity even after experimental timespan.

In summary, this study provides robust evidence that LDNM monotherapy with HFn-DOX is expected to remodel the therapeutic outcome of advanced metastatic BC compared to the drug alone and also to improve anthracycline therapies based on liposomal DOX, with a redefinition of the central role of DOX for solid malignancies under the new perspective of metronomic treatments. Further investigations are necessary to thoroughly elucidate the individual contributions of targeted therapy and neoangiogenesis inhibition in the strong enhancement of the antitumor efficacy of HFn-DOX. On the horizon after this study is the possibility of countless developments, one of which is a reappraisal of current clinical settings by combining low toxic LDNM regimens with administration of established antiangiogenic agents.

## MATERIALS AND METHODS

### Cell cultures and *in vitro* studies

Murine Bioware-Ultra 4T1-Luc2 cell line (4T1-L), used as model of BC cells, have been purchased in 2011 from Perkin Elmer, confirmed by IMPACT I PCR profiling by the source, and have been passaged for fewer than 6 months. 4T1-L were cultured in RPMI 1640 medium supplemented with 10% foetal bovine serum, 2 mM l-glutamine, penicillin (50 UI mL^−1^) and streptomycin (50 mg mL^−1^) at 37 °C in humidified atmosphere containing 5% CO_2_ and sub-cultured prior to confluence using trypsin/EDTA. 4T1 cells, were used for *in vitro* tests and orthotopically implanted at passages lower than 4 in female Balb/C mice to obtain the BC animal model.

Details of HFn-DOX production, cell binding, proliferation, death and DNA damage assays, intracellular localization by confocal laser scanning microscopy, are provided in the Supplementary Materials and Methods.

### Study design

The hypothesis was that HFn-DOX would exhibit higher antitumor efficacy and would induce minimal or negligible side effects compared to free drug and pl-DOX (Caelyx) in mice bearing strongly invasive and metastatic BC. HFn-DOX dose was set at 1.24 mg kg^−1^ DOX, about 1/7 of the average MTD dosage administered in 4T1 murine BC [26]. This tumor model was selected for its aggressiveness and spontaneous tendency to spread to multiple metastatic sites after orthotopic injection of luciferase-tagged cells. The endpoint of the *in vivo* experiments was defined at 21 days to appreciate the parametric differences in tumor growth, resistance onset and cardiotoxicity in living animals, while allowing us to operate in compliance with the National and European legislations that regulate animal experiments. The number of animals for each biodistribution, bioavailability, therapy and cardiotoxicity experiment was calculated with a power of at least 80 ± 5 %. Mice were randomized by primary tumor size before initiation of treatments. Dye-labeled HFn was first injected in tumor-bearing mice by tail vein, then targeting and biodistribution were assessed by live fluorescence imaging, while drug bioavailability was evaluated in healthy animals. Rodents were administered intravenously with placebo, DOX, pl-DOX or HFn-DOX at day 5, 9, 13 and 17, and monitored for 21 days during which tumor growth was followed by measurement of bioluminescence signal intensity (BLI) of 4T1-L cells after intraperitoneal injection of luciferin. BLI analyses were undertaken under standardized conditions to gain a quantitative estimation of live BC cells. Intermediate BLI values and mouse weights were determined before each administration. Collected BLI data were normalized to the mean tumor size calculated for all mice within each group at each time point. Animals were euthanized at day 21 to analyze resected tissues with the aim of determining the antitumor efficacy, anti-angiogenic activity and cardiotoxicity of DOX, pl-DOX and or HFn-DOX. Histopathology and immunohistochemistry were analyzed from blinded samples. Outliers were not excluded. All experiments were conducted under an approved protocol of the Italian Ministry of Health. Animals were cared for according to the guidelines of the Italian Ministry of Health (see the Supplementary Materials and Methods).

### *In vivo* experiments

Details of the preparation of orthotopic 4T1 model, tumor cell injection, tumor imaging, targeting and biodistribution, plasma half-life, drug accumulation at the tumor of DOX and HFn-DOX and antitumor *in vivo* efficacy of DOX, pl-DOX or HFn-DOX are provided in Supplementary Materials and Methods.

### *Ex vivo* analyses

Excised tumors were analyzed by fluorescence imaging and by confocal microscopy of cryosections to establish the HFn cellular targeting *in vivo*, immunofluorescence of dissociated tumor to assess DOX accumulation, immunohistochemistry to determine the CD31 and MDR-1 expression in endothelial and tumor cells, respectively, and Tumor TACS *In Situ* Apoptosis Detection kit to determine cellular apoptosis. Excised organs were analyzed by fluorescence imaging to establish the HFn biodistribution in non-target organs. Histopathology was performed on samples from liver, kidneys, spleen, heart, brain, gut and lung tissues. Kidney and liver functionality was assessed before and after the treatment. The size of cardiomyocytes extracted from resected heart tissues was measured after wheat germ agglutinin (WGA) fluorescence labeling. Isolated mitochondria from heart tissue samples were investigated by membrane potential and ultrastructural analysis of transmission electron micrographs; the extent of ROS in heart was assessed by glutathione assay. Details are reported in Supplementary Materials and Methods.

### Statistical analysis

Statistical analyses were conducted using two-tailed Student's *t*-test. All plots show mean values ± SE. All tests assumed normal distribution and the statistical significance threshold was set at *P*< 0.05

### Ethics statements

Investigation has been conducted in accordance with the ethical standards and according to the Declaration of Helsinki and according to national and international guidelines and has been approved by the authors' institutional review board.

## SUPPLEMENTARY MATERIALS FIGURES AND TABLES


