# Supplementary material for: Nanometronomic treatment of 4T1 breast cancer with nanocaged doxorubicin prevents drug resistance and circumvents cardiotoxicity
Source: Oncotarget. 2016 Dec 25;8(5):8383–96. doi: 10.18632/oncotarget.14204 (PMC5352408; doi:10.18632/oncotarget.14204)
Supplement: Supplementary file 1 [file oncotarget-08-8383-s001.pdf]

# Nanometronomic treatment of 4T1 breast cancer with nanocaged doxorubicin prevents drug resistance and circumvents cardiotoxicity

## SUPPLEMENTARY DATA

### HFn production

HFn nanocages were produced in *E. coli* by DNA recombinant technology and purified as previously described (22). HFn was labeled with fluoresceine isothiocyanate (FITC) or with Alexa $Fluor_{660}$ for *in vitro* and *in vivo* studies, respectively. HFn was filled in with DOX with a loading efficiency of 29 drug molecules per HFn. Quantification of DOX payload was undertaken by fluorescence measurements (22).

### Cell binding assay

Cells ($5 \times 10^5$) were incubated 2 h at 4 °C in flow cytometry tubes in the presence of 20 or 100 µg $mL^{-1}$ of FITC-labeled HFn. After incubation, cells were washed three times with PBS. Labeled cells were resuspended with 0.5 mL of phosphate-buffered saline (PBS, EuroClone) and analyzed by a Gallios flow cytometer (Beckman Coulter). 20000 Events were acquired for each analysis, after gating on viable cells, and a sample of untreated cells was used to set the appropriate gates.

### Cell proliferation assay

Cells were cultured on a 96 multiwell dish at a density of 5000 cells $cm^{-1}$. Then, cells were incubated with different amounts of the molecules to be tested. At the indicated time points (24, 48, 72 h), cells were washed with PBS and then incubated for 3 h at 37 °C with 0.1 mL of [3-(4,5-dimethylthiazol-2-yl)-5-(3-carboxymethoxyphenyl)-2-(4-sulfophenyl)-2H-tetrazolium salt (MTT) stock solution previously diluted 1:10 in DMEM medium without phenol red. After incubation, MTT solubilizing solution (0.1 mL) was added to each well to solubilize the MTT formazan crystals (Promega). Absorbance was read immediately using a testing wavelength of 570 nm and a reference wavelength of 620 nm. The results are expressed as means ± standard error (SE) of six individual experiments.

### Cell death assay

Cells were cultured on a 12 multi-well dish until sub-confluence. Then, cells were incubated 3 h and 24 h at 37 °C in the presence of different amounts of DOX or HFn-DOX. After incubation, cells were washed twice with PBS and treated for FACs analysis according to Annexin V-PE-Cy5 Apoptosis Detection Kit manufacturer's protocol (BioVision). Cells were analyzed within 1 h on a Gallios flow cytometer (Beckman Coulter). 20000 Events were acquired for each analysis, after gating on cells.

### Western blot

Briefly, 4T1-L cells were cultured in a 6-wells plate, lysed with 200 µL lysis buffer (20 mM Tris HCl pH 7.6, 150 mM NaCl, 1 mM EDTA, 1% Triton X-100, 1% glycerol, 1 mM $Na_3VO_4$, 10 mM NaF, Protease Inhibitor Cocktail, 1 mM PMSF). Total protein in lysate was quantified using the Coomassie Plus Protein Assay Reagent (Thermo Fisher Scientific) with Bovine Serum Albumin (BSA) as standard protein. Approximately 30 µg of protein from each sample were separated by SDS-PAGE using 12% (v/v) polyacrylamide gels and then transferred onto PVDF membrane. The membrane was blocked in 5% fat-free milk powder in PBS with 0.1 % Tween 20 for 1 h. The membrane was incubated overnight with mouse-monoclonal antibody against P-gp (cod. Ab170904; Abcam) at 1:1000 dilution or a mouse monoclonal antibody anti-α-tubulin (Sigma) at 1:1000 dilution in 5% fat-free milk powder in PBS with 0.1 % Tween 20 for 1 h. The membrane was washed three times with PBS with 0.1 % Tween 20 and reacted 1 h with the secondary antibody anti-mouse conjugated with horseradish peroxidase (1:5000; Abcam) for 1 h. The bound antibody was revealed using ECL star reagent (Euroclone) and the chemoluminescence signal was detected using the Chemidoc System (Biorad).

### Confocal laser scanning microscopy

Cells were cultured on collagen pre-coated cover glass slides until sub-confluence and incubated with HFn for different time periods. To evaluate internalization, FITC labeled HFn (100 µg $mL^{-1}$) were incubated with cells for 15 min, 1, 3 and 48 h at 37 °C. DNA damage was assessed 24 h after incubation DOX free or encapsulated in HFn cage. To maintain a good degree of live cells we have worked with the DOX dosage of 0.01 µM. In order to evaluate nuclear DOX release cells were incubated for 3 h at 37 °C with DOX free or encapsulated in HFn at the concentrations of 0.1 or 1 µM. At the end of incubation with nanoparticles, cells were washed with PBS, fixed

for 5 min with 4% paraformaldehyde (Sigma) and then treated for 5 min with 0.1% Triton X-100 (Sigma). A blocking step was performed for 1 h at room temperature with a solution containing 2% bovine serum albumin (BSA, Sigma), 2% goat serum (Euroclone) and 0.2 μg $mL^{-1}$ DAPI (4',6-diamino-2-phenylindole; Invitrogen) in PBS. Golgi apparatus, lysosomes, early and recycling endosomes were stained respectively with Golgi marker 130 (GM-130; at a 1:100 dilution; clone 35; BD Biosciences), Cathepsin D (CatD; 1:50; clone BC011; Calbiochem), Early Endosomes Antigen-1 (EEA-1; 1:1000; clone 14; BD Biosciences), Transferrin (Tf; 1:100; clone 5G2; Abcam) antibodies by incubating 2 h at RT and revealed by a AlexaFluor 546-conjugated antibody against murine IgGs (Invitrogen) at a 1:300 dilution by incubating for 2 h at RT in PBS, 2% BSA, 2% goat serum. Double strand break were stained with γH2A.X antibody (1:1000 dilution; Abcam) by incubating 2 h at room temperature and revealed by AlexaFluor 546-conjugated antibody against rabbit IgGs (Invitrogen) at a 1:300 dilution by incubating for 2 h at room temperature in PBS, 2% BSA, 2% goat serum. Microscopy analysis was performed with a Leica SPE microscope confocal system equipped with laser excitation lines 405 nm, 488 nm, 514 nm and 633 nm. Images were acquired with 63× magnification oil immersion lenses at 1024×1024 pixel resolution. DOX was revealed exciting the sample with 488 nm laser line and acquiring emitted signal from 550 nm to 600 nm (magenta). The signal represented in green color is the emission in the acquiring window between 520 to 545 nm, which correspond to the fluorescence signal of a degradation product of DOX, as previously reported (22).

## Production of orthotopic 4T1-L tumor model

For the *in vivo* experiments, 8-week old female Balb/C mice purchased by Charles River Laboratories (Calco, Italy), were maintained in a fully equipped facility, housed in single cages, fed ad-libitum and observed daily. Animals were anesthetized by intraperitoneal injection of 250 mg $kg^{-1}$ of avertin and were handled and euthanized according to ethical guidelines. Mice were used in accordance with an experimental protocol subjected to the direct approval of the Italian Ministry of Health. 4T1-L cells, grown as described above, were injected in 8-week old female Balb/C mice. All tumor injections were done 2 days after abdominal trichotomy. 4T1-L ($1×10^5$ cells for each animal) were suspended in cold serum-free RPMI 1640 growth medium and injected into mammary fat pad. Animals were observed and tumor formation was recorded at least thrice per week. For biodistribution studies, the tumors were allowed to grow for 7 days, at which time they reached a size of 0.8 $cm^3$ approximately, while for efficacy studies, the tumors were allowed to grow only for 5 days.

## Tumor targeting and biodistribution of AF660 labeled HFn nanovector

Balb/C mice bearing 4T1-L cells were immobilized in a restrainer (2 biological instrument) to be injected in the tail vein with AF660-labeled HFn void nanovectors (5 mg $kg^{-1}$ body weight). Epifluorescence imaging was performed at 1, 2, 4, 24 and 48 h post-injection by placing the anesthetized animals in a IVIS Lumina II imaging system (Perkin Elmer) at 37 °C. Images were acquired with a 680 nm emission filter while excitation was scanned from 570 to 640 nm, and mice autofluorescence was removed by spectral unmixing. Bioluminescence (BLI) images were acquired 5 min after luciferin peritoneal injection (150 μg $kg^{-1}$, Perkin Elmer) with an exposure time of 5 s. The instrument setup for BLI acquisition is Fstop 4 and medium binning. After *in vivo* acquisitions, mice were sacrificed and urine were collected from the bladder. Dissected tumors and organs (liver, kidneys, spleen, heart, brain and lungs) were analyzed in IVIS system, as described above for the whole animals. Fluorescence intensity of equal amounts of urine was analyzed in a GloMax Multi Detection System (Promega), to evaluate the presence of HFn.

## *Ex-vivo* analysis of tumor cryosections

4T1-L tumors were isolated and fixed in 4% paraformaldehyde solution for 3 h, washed in PBS and embedded in OCT for freezing in liquid nitrogen. 10 μm thick tumor cryosections were air dried at room temperature for 1 h, rinsed with PBS and, after 5 min of permeabilization at room temperature with 0.1% Triton X-100 in PBS, counterstained with DAPI (diluted 1:1500 in PBS) for 20 min at room temperature. Microscopy analysis of cryosections was performed with a Leica TCS SPE confocal microscope (Leica Microsystems). Images were acquired with 40× magnification oil immersion lenses at 1024×1024 pixel resolution.

## Plasma half-life

To determine plasma half-life of nanoformulated DOX in comparison to free drug, HFn-DOX or DOX (1.24 mg DOX $kg^{-1}$ or 12.4 mg DOX $kg^{-1}$) were intravenously (i.v.) injected into healthy Balb/C mice. At selected time points blood was collected in EDTA coated tubes by collection from the retro-orbital plexus. Plasma was prepared by centrifugation at 1500 ×g for 15 min. DOX was extracted as reported (29) and the content was measured by a spectrofluorometer (Horiba; $\lambda_{ex}$ = 500 nm; $\lambda_{em}$ = 550 nm). The DOX amount in plasma was quantified using a standard curve previously obtained extracting known amounts of DOX from plasma. The data are the

mean ± SE of samples collected from at least 3 different mice.

### Tumor dissociation

Balb/C mice orthotopically implanted with 4T1-L cells, as reported above, were anesthetized and injected into the lateral tail vein with DOX or HFn-DOX (1.24 mg $kg^{-1}$). Control mice were injected with sterile saline solution. Mice were euthanized (n = 2 mice/group); tumors were excised at 2 h after injection and dissociated using the Mouse Tumor Dissociation kit (Miltenyi Biotech). Dissociated cells were seeded on glass slide with the Cytospin Centrifuge (Thermo Scientific). The resulting samples were fixed with 4% paraformaldehyde and processed for immunofluorescence as described above.

### Doxorubicin quantification in tumors

Balb/C mice orthotopically implanted with 4T1-L cells, as reported above, were anesthetized and injected into the lateral tail vein with DOX or HFn-DOX (1.24 mg $kg^{-1}$; n = 24 mice/group). 1, 2, 4 and 24 h after injection mice were euthanized (n = 3 mice/group) and tumors were collected. Tumor were weighted, homogenized in water (10% w/v) with potter (Glas-Col homogenizer) and DOX was extracted as reported (29). The drug content was measured by a spectrofluorometer (Horiba; $\lambda_{ex}$ = 500 nm; $\lambda_{em}$ = 550 nm) and quantified using a standard curve previously obtained extracting known amounts of DOX from tumor homogenates. The data are the mean ± SE of samples collected from at least 3 different mice.

### *In vivo* efficacy

Balb/C mice were orthotopically implanted with 4T1-L cells. Five days after implant, mice were randomly divided into three experimental groups and anesthetized. Five min after intraperitoneal injection of luciferin (150 µg $kg^{-1}$, Perkin Elmer) bioluminescence (BLI) images were acquired (IVIS Lumina II, Perkin Elmer, exposure time of 5 sec, Fstop 4 and medium binning). The region of interest (ROI) was drawn by fixing the lower value of the scale at 50. The number of photons emitted/sec in the ROI were used to measure tumor volume. Then, mice were injected into the lateral tail vein with DOX, pl-DOX and HFn-DOX (1.24 mg $kg^{-1}$) or with sterile saline solution (placebo). Therapeutic treatment was repeated at day 9, 13 and 17 after 4T1-L cells implants and BLI signal of tumors were measured before every treatment. At day 21 tumor BLI were measured and mice were euthanized. Tumors were excised, weighed, fixed in 10% formalin and embedded in paraffin for immunohistochemistry. Liver, kidneys, lungs, spleen, gut and hearts (half) were excised (n = 3/group), fixed in 10% formalin and embedded in paraffin for histopathological analysis. Hearts (n = 9/group) were excised, frozen in liquid nitrogen and stored at –80 °C for further evaluations.

### Immunohistochemistry

Three micrometer thick paraffin-embedded tissues (n = 5/group) were cut, deparaffinized in xylene, and rehydrated in ethanol. Microwave oven pretreatment was performed (pH 8.0, EDTA buffer). Immunohistochemistry was performed using a polyclonal antibody anti-CD31 (1:200 dilution, DakoCytomation, 2 h incubation) and a rabbit monoclonal antibody anti-MDR-1 (1:100 dilution, Abcam, 2 h incubation). The reaction was revealed by means of supersensitive nonbiotin detection system (BioGenex) and diaminobenzidine as chromogen. The number of vessels was counted on 10 fields/sample, while quantification of MDR-1 expression was performed by ImagePro Plus software on 5 fields/sample. Magnification 40× (vessels number); magnification 20× (MDR-1 expression).

### Apoptosis assay

Paraffin embedded tumor sections (n = 6/group) were treated with the Tumor TACS In Situ Apoptosis Detection kit (Trevigen) according to manufacturer's protocols, which labels apoptotic nuclei in paraffin-embedded tissue sections. Apoptotic nuclei were counted on 10 squares/sample (n = 6/group). Magnification 20×.

### Histopathological analysis

Liver, kidneys, spleen, heart, brain, gut and lung samples obtained from Balb/C mice (n = 3/group) were fixed in 10% buffered formalin for at least 48 h and embedded in paraffin. Three micrometer sections were cut, stained with hematoxylin and eosin, and examined in a blinded manner. For cardiomyocyte cross-dimensions analysis, heart sections were deparaffined by washing sections with xylene for 30 min at room temperature. Then, sections were washed with ethanol 100%, 95%, 90%, 85%, 80%, 70% and thrice with water, fixed with 4% paraformaldehyde in PBS for 1 h and stained 1 h with Wheat Germ Agglutinin coupled to Alexa Fluor 488 (1:200; Wheat Germ Agglutinin, Alexa Fluor488-conjugate, Invitrogen). Tissue were washed with PBS and mounted with Prolong Gold (Invitrogen). Slides stained with wheat germ agglutinin were acquired at using fluorescence microscope (Nikon Eclipse 80i) at magnification 40×. Image dimensions 2560×1920 pixels. Cardiomyocyte areas were quantified using ImageJ software.

## Ultrastructural analysis (TEM)

Small portions of hearts (n = 3/group) samples were fixed in 2.5% glutaraldehyde in 0.1 M phosphate buffer, pH 7.2, for 2 h. After one rinsing with PBS, specimens were post-fixed in 1.5% osmium tetroxide for 2 h, dehydrated by 50, 70, 90, and 100% EtOH, and embedded in epoxy resin (PolyBed 812 Polysciences Inc.). Ultrathin sections were cut with an ultramicrotome (Ultracut E (Reichert-Jung)), stained with uranyl acetate and lead citrate and examined by means of a transmission electron microscope (TEM, Tecnai Spirit (FEI)). For mitochondria quantification at least 9 images/group were taken at 4200× magnification. Three mice were used for each experimental condition. Mitochondrial morphometric measurements were performed using ImageJ software on at least 10 images/group acquired at 11.500× magnification measuring the area of at least 100 mitochondria/sample. The percentage of area occupied by mitochondrial cristae was measured by ImageJ software imposing a threshold value of 118.

## Mitochondria isolation and evaluation of membrane potential

Heart tissue (50 mg) were homogenized in a potter (Glas-Col) in 2 mL of Mitochondria Isolation Buffer supplemented with 1 mg $mL^{-1}$ Bovine Serum Albumin (225 mM mannitol, 75 mM sucrose, 10 mM HEPES, 10 mM EDTA) and centrifuged for 10 min at 1000 ×g. Supernatants were centrifuged 10 min at 12000 ×g to harvest crude mitochondria pellet. Pellets were washed thrice in Mitochondria Isolation Buffer. Protein concentrations were determined using Bradford assay. Mitochondria membrane potential was determined in isolated mitochondria using JC-1 reagent (Life Technologies) according to the manufacturer's protocols. Briefly, mitochondria were labeled with 1.5 mg JC-1/mg protein and acquired in flow cytometry (Cytoflex, Beckman Coulter). Samples were excited using a 488 nm laser source. The signal of JC-1 monomers was captured by FL1, while the signal of JC-1 aggregates was collected by FL2. The mitochondrial membrane potential is represented by the ratio between the median fluorescence intensity of FL2 and FL1 normalized on protein concentration.

## Glutathione assay

The measurement of reactive oxigen species (ROS) was performed by quantifying reduced glutathione (GSH) in hearts. 50 mg of heart tissue was washed in heparin (10000 U $mL^{-1}$), homogenized in PBS supplemented with 2 mM EDTA (1 mL) with a potter (Glas-Col) and centrifuged for 10 min at 10000 ×g. The GSH levels of the collected supernatants were assessed using a luminescence-based GSH-Glo Glutathione assay (Promega) according to the manufacturer's protocol.

## Assessment of kidney and liver functionality

Kidney and liver functionality were assessed by measuring the amount of urea, creatinine, AST and ALT in plasma (n = 3/group) before (day 5) and after (day 21) the treatment with placebo, DOX (1.24 mg $kg^{-1}$), pl-DOX (1.24 mg $kg^{-1}$ DOX) and HFn-DOX (1.24 mg $kg^{-1}$ DOX). Quantifications were performed according to the manufacturer's protocols using the following kits: QuantiChrom™ Urea Assay Kit, QuantiChrom™ Creatinine Assay Kit, EnzyChrom™ Aspartate Transaminase Assay Kit and EnzyChrom™ Alanine Transaminase Assay Kit (BioAssay Systems).

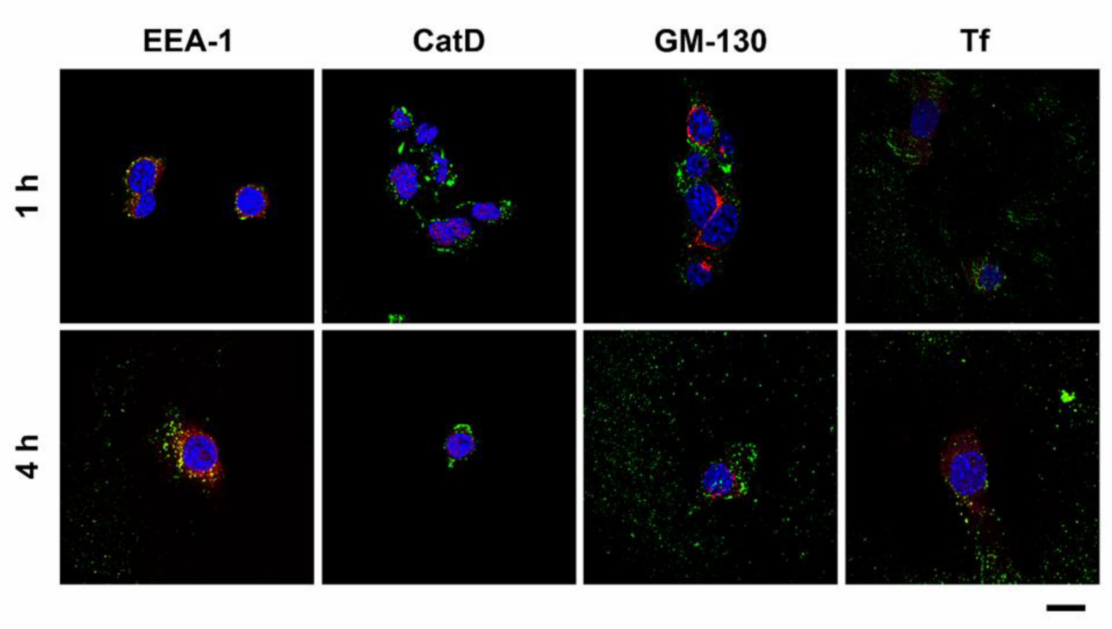


**Supplementary Figure 1: Subcellular analysis of HFn interaction with 4T1 BC cells reveals early intracellular localization in endosomes: colocalization with markers of endocytic pathway.** Confocal microscopy merged images of 4T1-L cells, incubated for 1, and 4 h at 37 °C with 100 µg $mL^{-1}$ of HFn (green). Early endosomes, lysosomes, Golgi and recycling endosomes were recognized with primary antibodies against early endosome antigen-1 (EEA-1), lysosomal protein Cathepsin D (CatD), Golgi marker 130 (GM-130) and Transferrin (Tf) as recycling endosome marker, respectively, and labeled with an anti-mouse secondary antibody conjugated with Alexa Fluor 546 (red). Nuclei were stained with DAPI (blue). Scale bar: 10 µm.

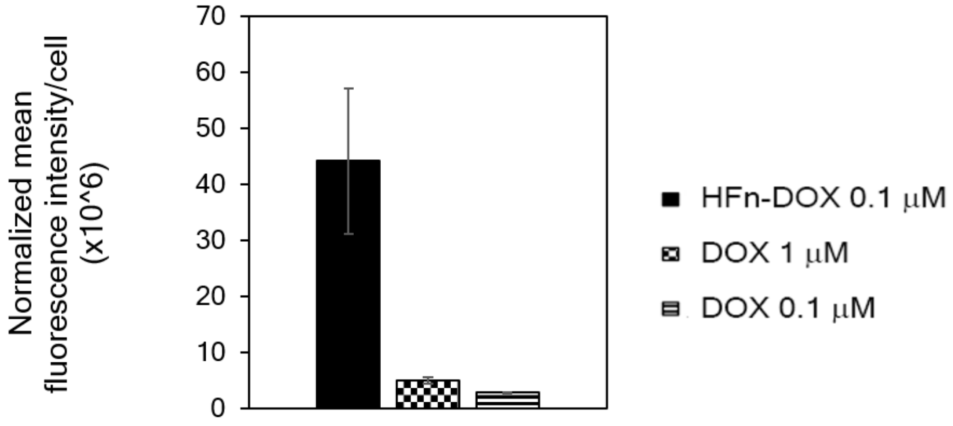


| *P*-value | *vs* DOX 0.1 μM | *vs* DOX 1 μM |
|---|---|---|
| HFn-DOX 0.1 μM | 0,005395653 | 0,008592103 |

**Supplementary Figure 2: Quantification of DOX nuclear accumulation *in vitro* upon treatment with DOX or HFn-DOX.** Quantification of the mean fluorescence signal of DOX in the nucleus. Reported values are mean of measurements performed with ImageJ software on nuclei of twenty different cells normalized with respect to their area.

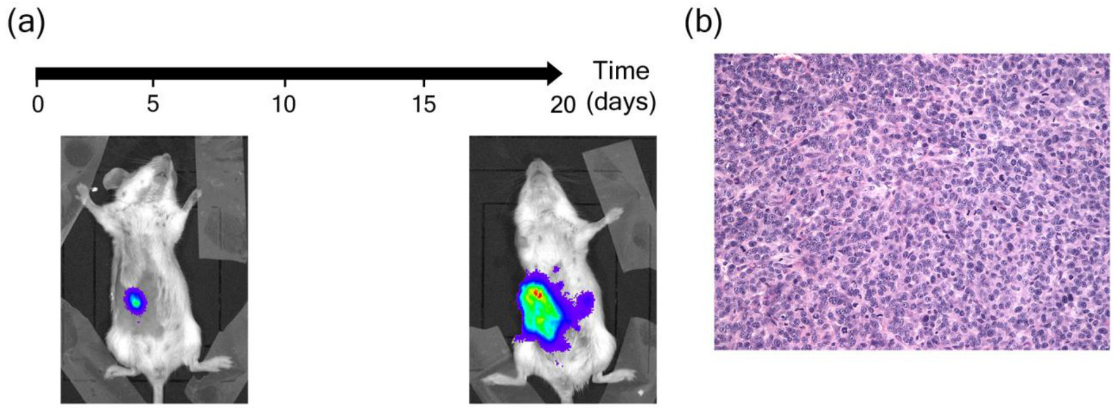


**Supplementary Figure 3: Development of 4T1-L breast cancer model: tumor growth and metastasis. a.** Balb/C female mice were injected in the mammary fat pad with $10^5$ cells/mouse. Bioluminescence intensity emitted upon intraperitoneal injection of luciferin was acquired 5 and 20 days after tumor implantation. **b.** Hematoxylin-eosin staining of 4T1-L tumor section.

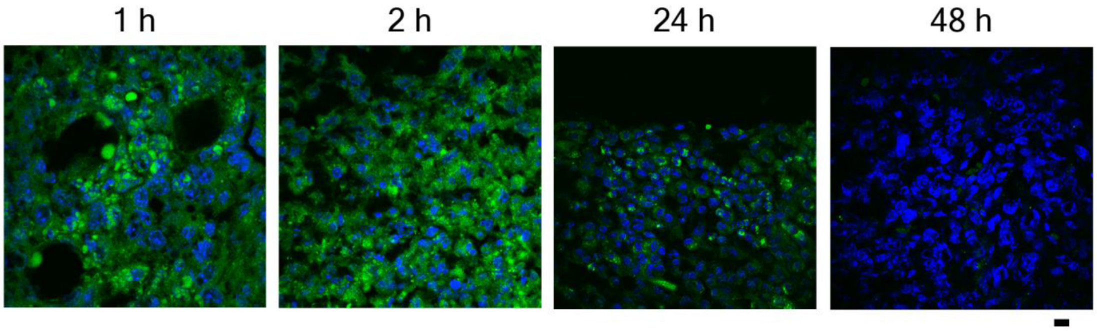


**Supplementary Figure 4: HFn nanoparticle localization in tumor cryosections.** Confocal microscopy merge images of cryosections obtained from 4T1-L tumors excised 1, 2, 24 and 48 h after HFn (green) intravenous injection. Nuclei were stained with DAPI (blue). Scale bar: 10 µm.

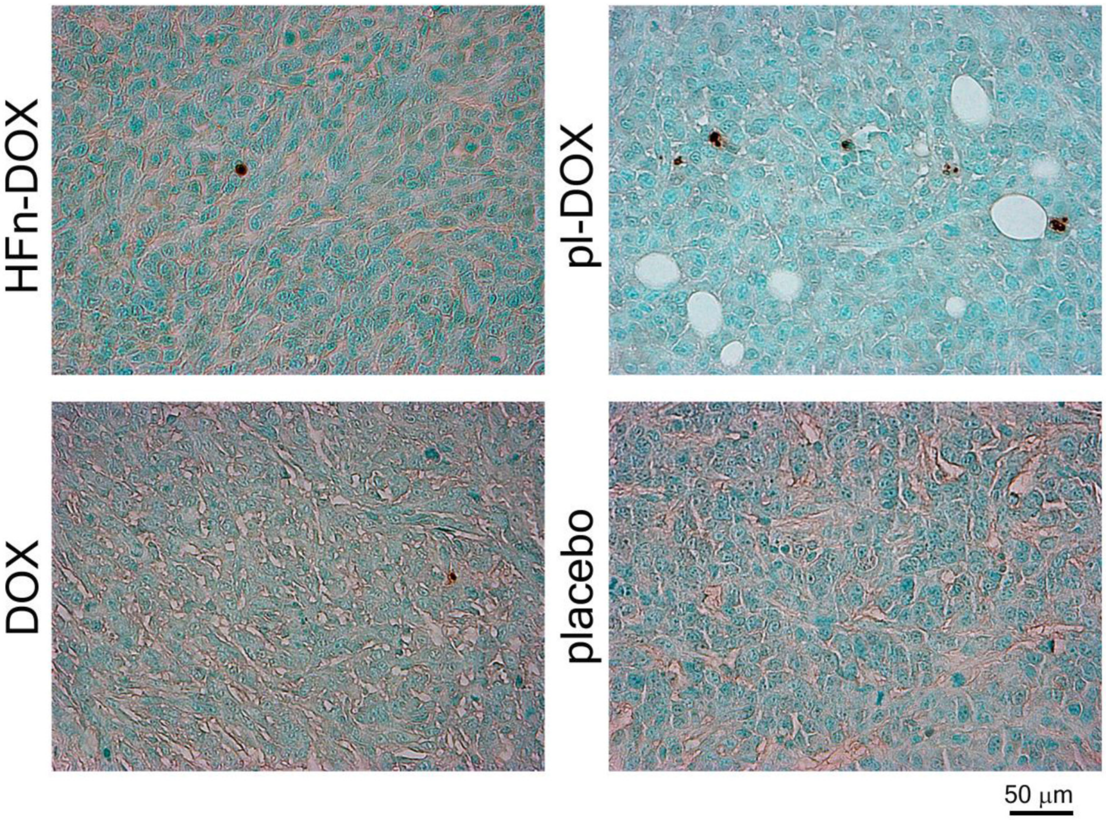


**Supplementary Figure 5: Immunohistochemistry of Apoptosis assay in tumor.** Representative images of tumors excised at day 21 (n = 6/group) from mice treated with placebo and 1.24 mg kg$^{-1}$ of DOX, pl-DOX or HFn-DOX. Samples were fixed with formalin, embedded in paraffin and histological slides were stained with Tumor TACS In Situ Apoptosis Detection kit to reveal apoptosis.

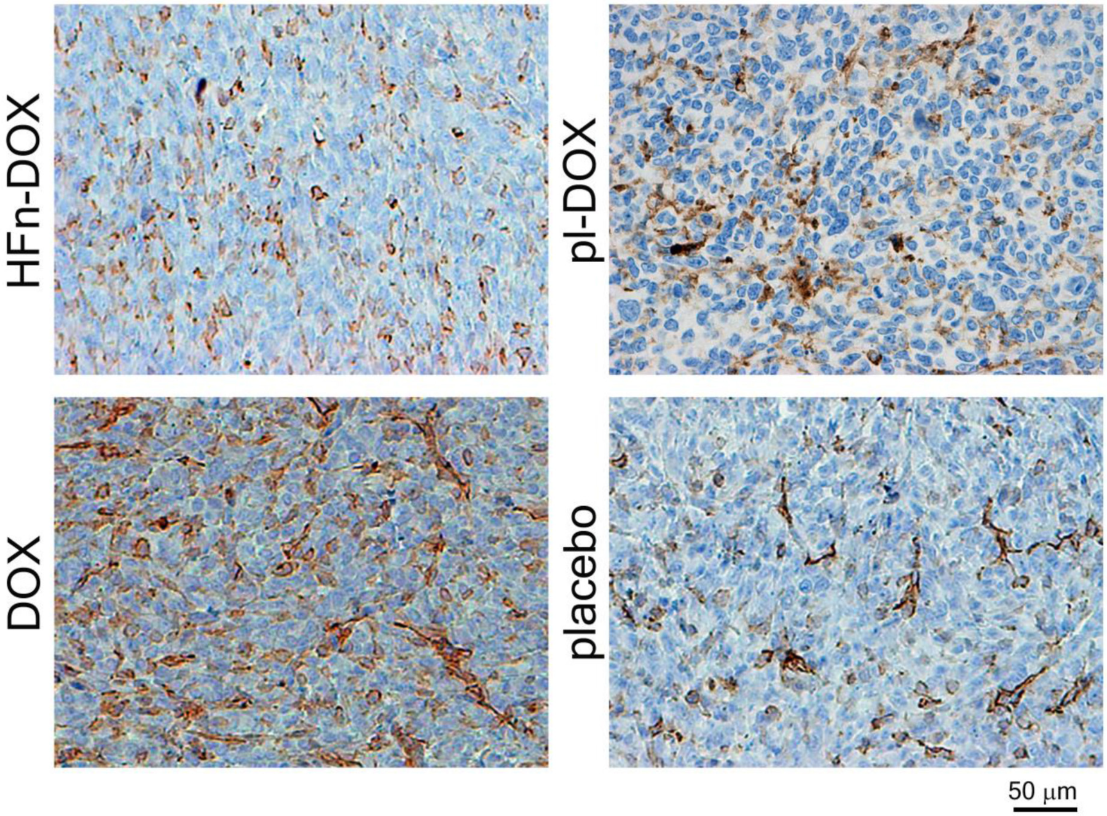


**Supplementary Figure 6: Immunohistochemistry of CD31 expression in tumor.** Representative images of tumors excised at day 21 (n = 5/group) from mice treated with placebo and 1.24 mg kg$^{-1}$ of DOX, pl-DOX or HFn-DOX. Samples were fixed with formalin, embedded in paraffin and histological slides were subjected to immunohistochemistry staining with the CD-31 antibody to reveal tumor angiogenesis.

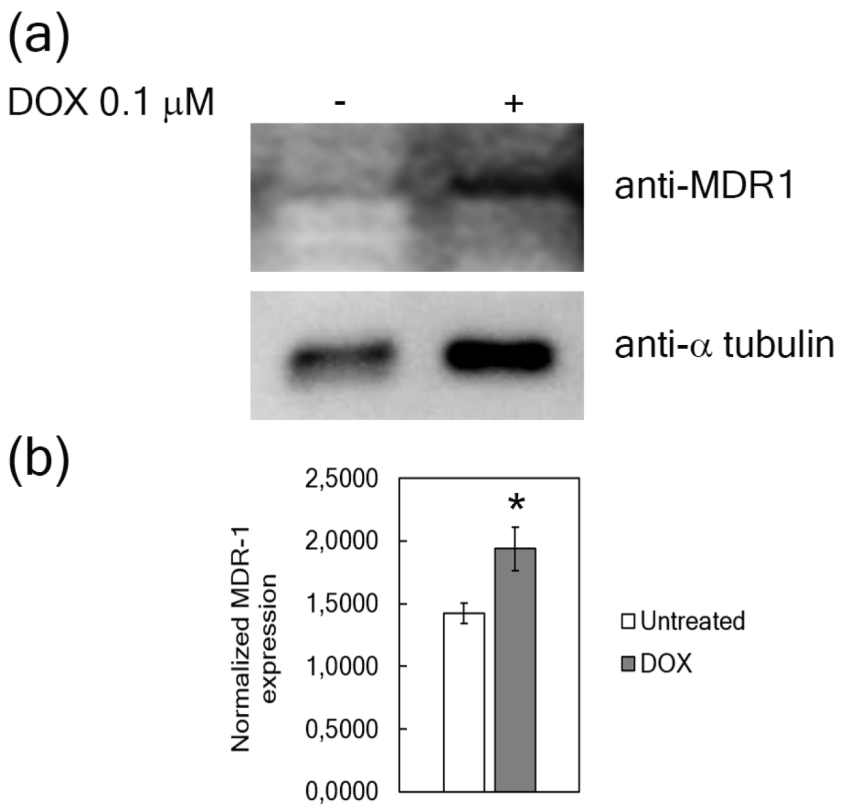


**Supplementary Figure 7: Basal and DOX-induced expression of MDR-1 in 4T1-L cells. a.** 4T1-L cells incubated for 72 h with or without DOX 0.1 μM were lysed. Whole cell extracts were loaded on SDS-PAGE application buffer, electrophoresed and immunoblotted using either anti-MDR-1 and anti-αtubulin antibodies. **b.** Quantification of the relative intensities of immunoblotted bands performed using ImageJ. Reported values represent the mean ± SE (n = 3). *$P<0.05(0.026)$.

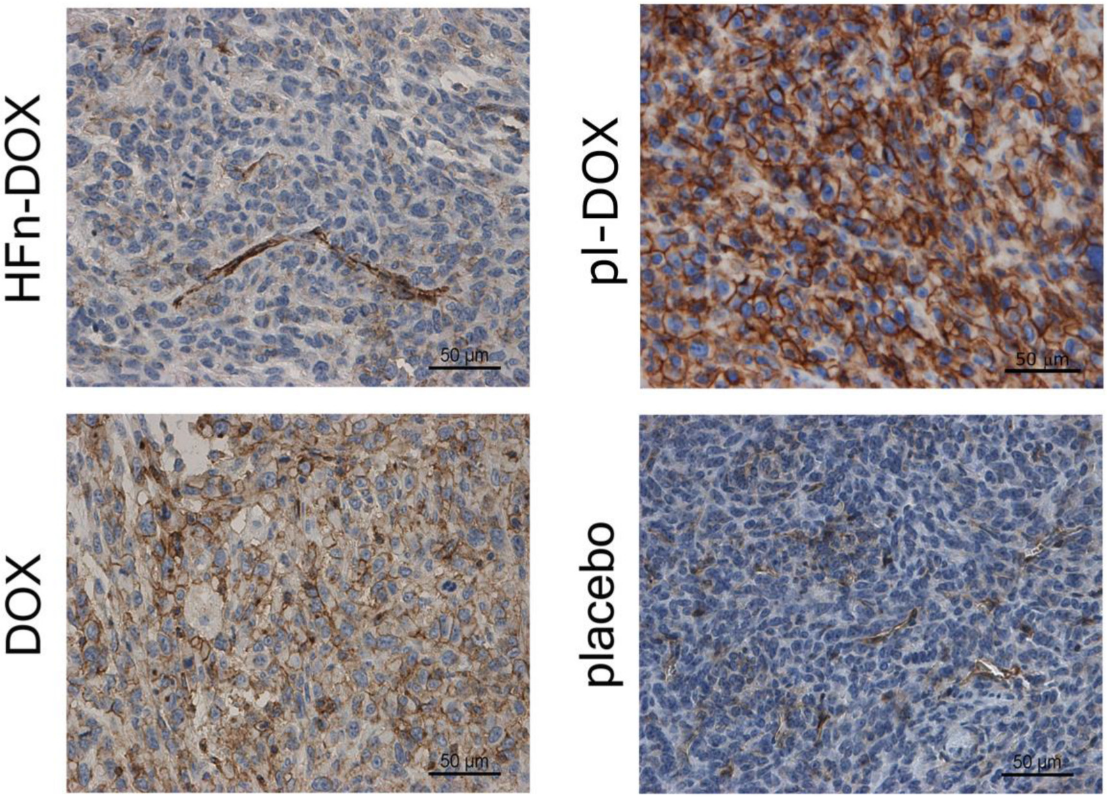


**Supplementary Figure 8: Immunohistochemistry of MDR-1 expression in tumor.** Representative images of tumors excised at day 21 (n = 5/group) from mice treated with placebo and 1.24 mg $kg^{-1}$ of DOX, pl-DOX or HFn-DOX. Samples were fixed with formalin, embedded in paraffin and histological slides were subjected to immunohistochemistry staining with the rabbit monoclonal antibody anti-MDR-1 to reveal MDR-1 overexpression. Scale bar = 50 µm.

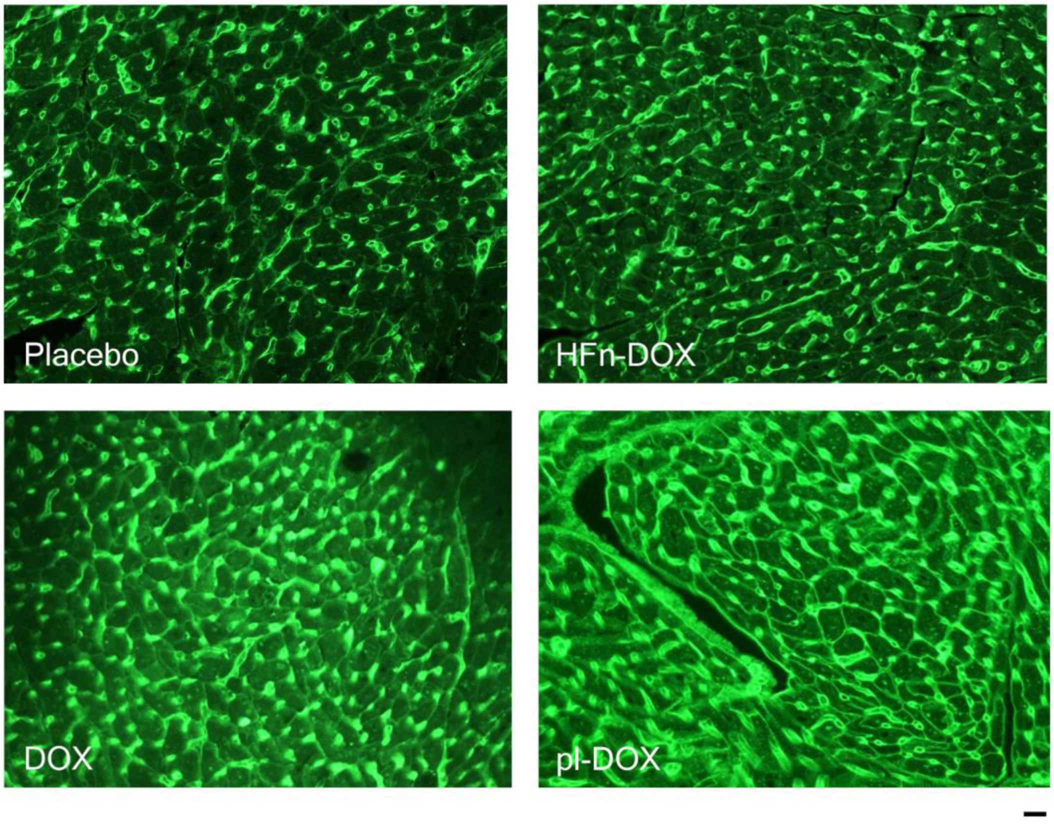


**Supplementary Figure 9: Fluorescence images of WGA-labeled cardiomyocytes membrane.** Representative fluorescence images of hearts excised at day 21 (n = 3/group) from mice treated with placebo or with 1.24 mg $kg^{-1}$ of DOX, pl-DOX or HFn-DOX. Samples were fixed with formalin, embedded in paraffin and histological slides were stained with FITC-conjugated Wheat Germ Agglutinin.

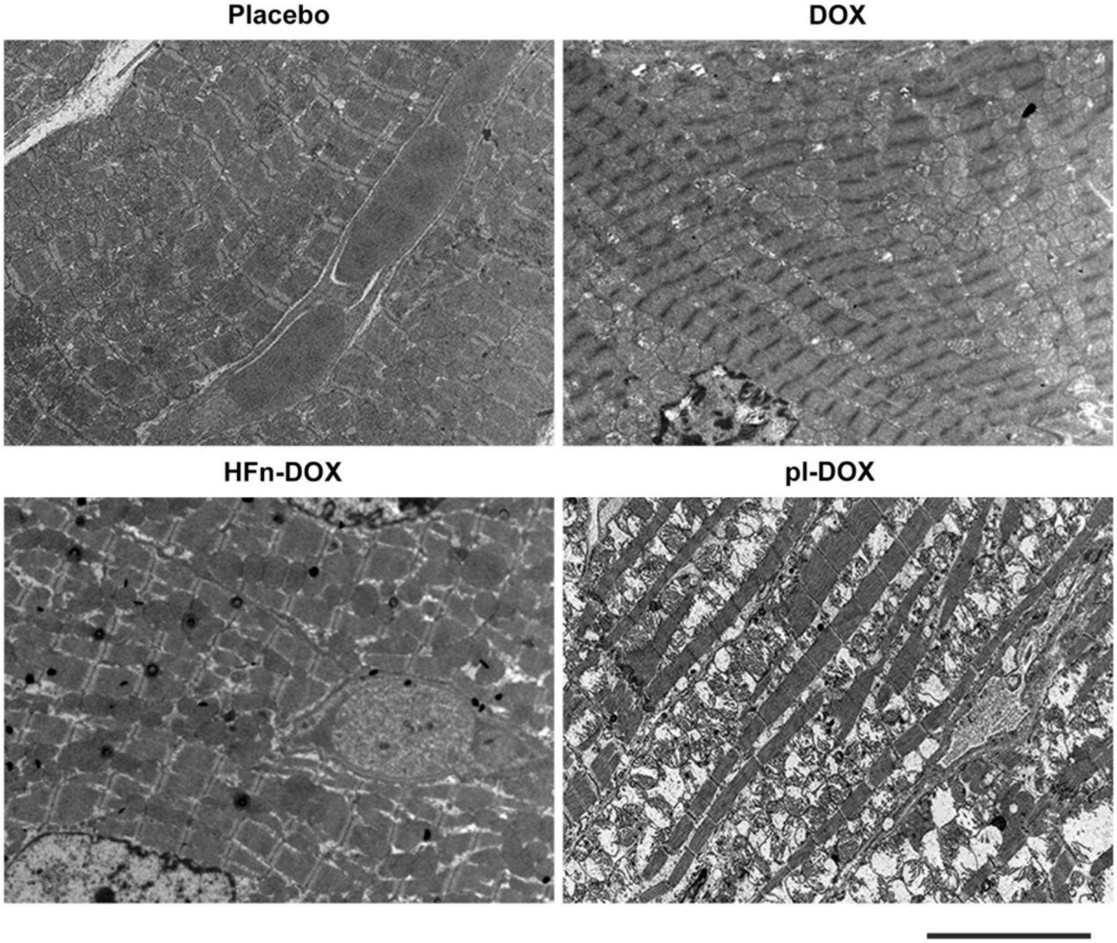


**Supplementary Figure 10: Ultrastructural evaluation of heart tissue sections after LDNM chemotherapy cycle.** Representative TEM images of hearts excised at day 21 (n = 3/group) from mice treated with placebo and with 1.24 mg $kg^{-1}$ of DOX, pl-DOX or HFn-DOX. TEM images of ultrathin heart sections of cardiac tissues have been acquired at 4200× magnification. Scale bar: 5 µm.

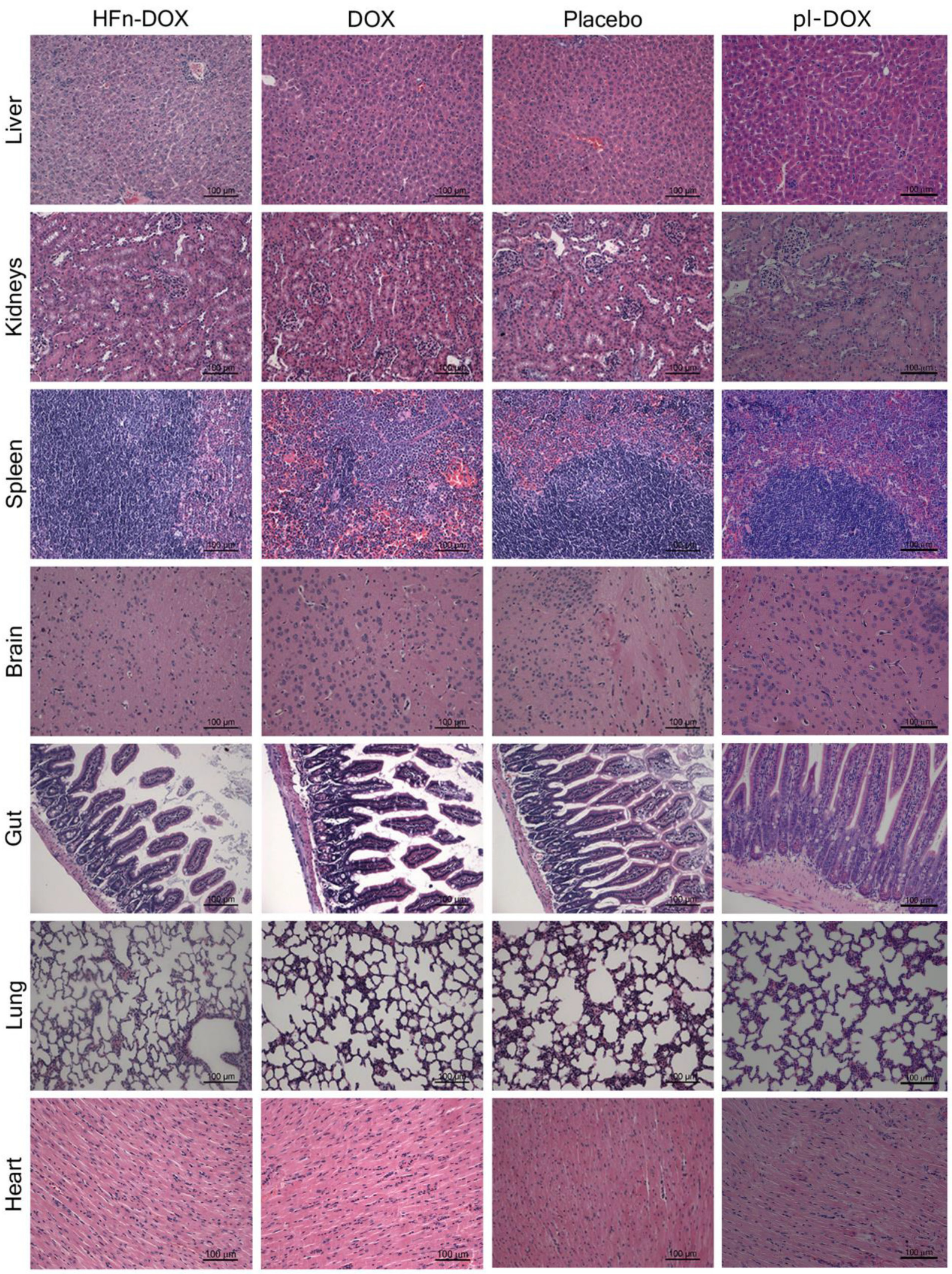


**Supplementary Figure 11: Histopathology of liver, spleen, kidneys, lungs, heart, gut and brain after LDNM treatment.** Histopathological analysis of tissue samples. No histological lesions were observed in liver, spleen, kidneys, lungs, heart, gut and brain. Hematoxylin-eosin staining. Magnification 20×.

**Supplementary Table 1: Impact in reduction of metastatic spread upon LDNM treatment with HFn-DOX**

| | *% metastatic tumors* | *P-value vs. CTRL* |
|---|---|---|
| HFn-DOX | 8.89 | 0.035 |
| DOX | 31.67 | 0.68 |
| pl-DOX | 10 | 0.09 |

**Supplementary Table 2: Liver functionality of mice treated with HFn-DOX, pl-DOX and DOX before and after LDNM treatment**

| | | Pre-treatment | Post-treatment |
|---|---|---|---|
| AST (U $L^{-1}$) | placebo | 129,61±22,44 | 148,52±37,12 |
| | HFn-DOX | 126,53±0,56 | 125,44±42,00 |
| | DOX | 144,57±6,92 | 161,74±44,30 |
| | pl-DOX | 160,60±10,76 | 188,66±17,32 |
| ALT (U $L^{-1}$) | placebo | 54,75±6,15 | 64,20±15,10 |
| | HFn-DOX | 52,73±17,87 | 44,11±8,91 |
| | DOX | 50,55±7,90 | 49,03±17,28 |
| | pl-DOX | 18,79±7,28 | 20,24±12,42 |

Reference values of healthy female Balb/C mice of 8-10 weeks are 54-298 U $mL^{-1}$ for AST, while 15-84 U $mL^{-1}$ for ALT.

**Supplementary Table 3: Kidney functionality of mice treated with HFn-DOX and DOX before and after LDNM treatment**

| | | Pre-treatment | Post-treatment |
|---|---|---|---|
| Urea (mg $dL^{-1}$) | placebo | 53,62±2,56 | 80,95±2,52 |
| | HFn-DOX | 44,49±2,03 | 68,73±7,46 |
| | DOX | 50,72±4,80 | 58,41±4,58 |
| | pl-DOX | 81,71±5,09 | 80,81±7,38 |
| Creatinine (mg $dL^{-1}$) | placebo | 4,52±2,41 | 9,09±0,83 |
| | HFn-DOX | 5,24±0,54 | 6,89±1,20 |
| | DOX | 3,82±0,43 | 8,90±0,68 |
| | pl-DOX | 2,99±0,12 | 2,46±0,28 |

**Supplementary Table 4: Collected *P* values from *in vitro* experiments**

| *In vitro* | Samples | *P* value *vs.* CTRL | *P* value *vs.* DOX |
|---|---|---|---|
| **Cell death assay** | 1 µM HFn-DOX 3 h | – | 0.003795908 |
| | 0.1 µM HFn-DOX 3 h | – | 1.79275E$^{-05}$ |
| | 0.01 µM HFn-DOX 3 h | – | 0.001304573 |
| | 1 µM HFn-DOX 24 h | – | 0.133690013 |
| | 0.1 µM HFn-DOX 24 h | – | 2.83929E$^{-05}$ |
| | 0.01 µM HFn-DOX 24 h | – | 0.539141176 |
| **Proliferation assay** | 1 µM HFn-DOX 24 h | 0.000375374 | 0.400961555 |
| | 0.1 µM HFn-DOX 24 h | 0.06957145 | 0.357486986 |
| | 0.01 µM HFn-DOX 24 h | 0.641045742 | 0.001100148 |
| | 1 µM HFn-DOX 48 h | 3.78807E$^{-08}$ | 0.000103979 |
| | 0.1 µM HFn-DOX 48 h | 0.044433198 | 0.178689131 |
| | 0.01 µM HFn-DOX 48 h | 0.640297852 | 0.424377741 |
| | 1 µM 72 HFn-DOX h | 1.09913E$^{-12}$ | 9.54285E$^{-08}$ |
| | 0.1 µM HFn-DOX 72 h | 6.53302E$^{-12}$ | 3.32216E$^{-06}$ |
| | 0.01 µM HFn-DOX 72 h | 2.26315E$^{-07}$ | 0.009195454 |

**Supplementary Table 5: Collected *P* values from DOX quantification in tumors**

| ***P*-values HFn-DOX *vs.* DOX** | **1 h** | **2 h** | **4 h** | **24 h** |
|---|---|---|---|---|
| | 0,096263612 | 0,67714472 | 0,132438528 | 0,589619741 |

**Supplementary Table 6: Collected *P* values from *in vivo* experiments**

| *In vivo* | Samples | *P* value *vs.* CTRL | *P* value *vs.* DOX | *P* value *vs.* pl-DOX |
|---|---|---|---|---|
| **Tumor progression** | HFn-DOX 1.24 mg $kg^{-1}$ | 0.001978 | 0.017452 | 1,84851E-05 |
| **Apoptotic cells** | HFn-DOX 1.24 mg $kg^{-1}$ | 0.075791258 | 0.768520967 | |
| **Angiogenesis** | HFn-DOX 1.24 mg $kg^{-1}$ | 0.002686863 | 8.88912E-12 | 4,87092E-16 |
| **MDR1 expression** | HFn-DOX 1.24 mg $kg^{-1}$ | 0.086933528 | 0.000538666 | 4,90051E-05 |
| **Cardiomyocyte area** | HFn-DOX 1.24 mg $kg^{-1}$ | 3,95944E-20 | 2.99986E-26 | 1,90291E-91 |
| **Mitochondria number** | HFn-DOX 1.24 mg $kg^{-1}$ | 0.665871359 | 2.19978E-10 | 7.35291E-07 |
| **Mitochondria area** | HFn-DOX 1.24 mg $kg^{-1}$ | 2.79881E-06 | 1.34402E-08 | 4.85707E-07 |
| **% mitochondrial cristae area** | HFn-DOX 1.24 mg $kg^{-1}$ | 6.06111E-30 | 2.78012E-81 | 6.1E-80 |
| **Membrane potential** | HFn-DOX 1.24 mg $kg^{-1}$ | 0,419124686 | 0,070359877 | 0,023382726 |
